# Applying atomistic neural networks to bias conformer ensembles towards bioactive-like conformations

**DOI:** 10.1186/s13321-023-00794-w

**Published:** 2023-12-21

**Authors:** Benoit Baillif, Jason Cole, Ilenia Giangreco, Patrick McCabe, Andreas Bender

**Affiliations:** 1 Yusuf Hamied Department of Chemistry, University of Cambridge, Lensfield Rd, Cambridge, CB2 1EW UK; 2 Cambridge Crystallographic Data Centre, 12 Union Road, Cambridge, CB2 1EZ UK; 3 Present Address: Exscientia plc, The Schrödinger Building, Oxford Science Park, Oxford, OX4 4GE UK

**Keywords:** Atomistic neural network, Conformer ensemble, Bioactive conformation, Rigid-ligand docking, Maximum common substructure

## Abstract

Identifying bioactive conformations of small molecules is an essential process for virtual screening applications relying on three-dimensional structure such as molecular docking. For most small molecules, conformer generators retrieve at least one bioactive-like conformation, with an atomic root-mean-square deviation (ARMSD) lower than 1 Å, among the set of low-energy conformers generated. However, there is currently no general method to prioritise these likely target-bound conformations in the ensemble. In this work, we trained atomistic neural networks (AtNNs) on 3D information of generated conformers of a curated subset of PDBbind ligands to predict the ARMSD to their closest bioactive conformation, and evaluated the early enrichment of bioactive-like conformations when ranking conformers by AtNN prediction. AtNN ranking was compared with bioactivity-unaware baselines such as ascending Sage force field energy ranking, and a slower bioactivity-based baseline ranking by ascending Torsion Fingerprint Deviation to the Maximum Common Substructure to the most similar molecule in the training set (TFD2SimRefMCS). On test sets from random ligand splits of PDBbind, ranking conformers using ComENet, the AtNN encoding the most 3D information, leads to early enrichment of bioactive-like conformations with a median BEDROC of 0.29 ± 0.02, outperforming the best bioactivity-unaware Sage energy ranking baseline (median BEDROC of 0.18 ± 0.02), and performing on a par with the bioactivity-based TFD2SimRefMCS baseline (median BEDROC of 0.31 ± 0.02). The improved performance of the AtNN and TFD2SimRefMCS baseline is mostly observed on test set ligands that bind proteins similar to proteins observed in the training set. On a more challenging subset of flexible molecules, the bioactivity-unaware baselines showed median BEDROCs up to 0.02, while AtNNs and TFD2SimRefMCS showed median BEDROCs between 0.09 and 0.13. When performing rigid ligand re-docking of PDBbind ligands with GOLD using the 1% top-ranked conformers, ComENet ranked conformers showed a higher successful docking rate than bioactivity-unaware baselines, with a rate of 0.48 ± 0.02 compared to CSD probability baseline with a rate of 0.39 ± 0.02. Similarly, on a pharmacophore searching experiment, selecting the 20% top-ranked conformers ranked by ComENet showed higher hit rate compared to baselines. Hence, the approach presented here uses AtNNs successfully to focus conformer ensembles towards bioactive-like conformations, representing an opportunity to reduce computational expense in virtual screening applications on known targets that require input conformations.

**Supplementary Information:**

The online version contains supplementary material available at 10.1186/s13321-023-00794-w.

## Introduction

Predicting the three-dimensional (3D) target-bound conformations of molecules is important in geometry-based virtual screening techniques, where the potential activity of millions of molecules is computationally assessed [1]. Virtual screening relies mostly on ligand-based methods such as pharmacophore searching [2, 3] or shape-based screening [4, 5], or structure-based methods such as docking experiments [6–8] that are performed by predicting bioactive (target-bound) conformations [9], and require input of one or multiple 3D conformations for each tested molecule. However, there is a theoretically infinite number of conformers for a molecule, while only one or a few bioactive conformations are obtained from crystallographic or cryogenic electron microscopy experiments.

Conformer generation methods produce a limited number (e.g., in the order of hundreds) of plausible conformations on the Potential Energy Surface (PES) [10–14]. Conformer generators are often evaluated in their ability to retrieve known bioactive conformations using a minimal number of generated conformers [15, 16]. Using a common threshold of atomic-root mean square deviation (ARMSD) lower than 1 Å, recent generators such as the CSD conformer generator [10] or OMEGA [11] retrieve a bioactive conformation for around 90% of ligands from the Platinum [15] dataset with a conformer ensemble size of 250. Most generated conformers of flexible ligands are not bioactive-like (e.g., all ligands where 250 or more conformers were generated in the Musafia and Senderowitz study [17] showed less than 30 bioactive-like conformations), and testing all conformers in virtual screening applications requires significant computational resources. Therefore, having a fast way to distinguish bioactive-like from non-bioactive-like conformations among generated conformers allows short-listing conformations and hence reducing time spent in virtual screening.

Previous works investigated if conformer energy thresholds can be identified to discriminate bioactive conformations of ligands: guided by the induced fit model [18], studies observed that bioactive conformations often bind with a conformation that is not located at the global potential energy surface minima [19, 20], due to conformational change occurring upon binding. While different studies used diverse energy computation methods, leading to conflicting results [21], recent works on high-quality ligand conformations showed that more than half of them have low strain energy, computed with quantum mechanics: Zivanovic et al. showed that 73% of bioactive conformations are found at a maximum of 3k_B_T (1.78 kcal/mol for T = 298 K) from a local minimum conformer [22], and Tong and Zhao found a median strain energy of 2.5 kcal/mol [23]. Hence, exploring a diverse low energy landscape seems to be adequate when aiming to obtain conformations relevant for binding to a protein.

The use of conformation energy and descriptors to separate bioactive from non-bioactive conformations has been explored in several works. Diller and Merz [24] analysed 65 protein-ligand complexes and showed that bioactive conformations are found more often among conformers with larger solvent accessible surface area (SASA), higher radius of gyration (RGyr) and lower number of internal interactions, suggesting that ligands are more likely to bind in extended conformations. Auer and Bajorath [25] showed that high strain energy was a good discriminator to retrieve mostly bioactive conformations in the conformer ensemble for 228 ligands of 18 protein targets. Musafia and Senderowitz [17] developed models using combinations of 2D and 3D descriptors of 71 ligands, showing that selecting conformations using binned values of SASA and the principal moment of inertia magnitudes were able to enrich bioactive-like conformations and impoverish (i.e., decrease the number of, as defined by the authors in cited publication) non-bioactive conformations in these selected ensembles. Surprisingly, none of the energy terms computed were selected by the best models for bioactive-like conformation enrichment. The authors also published a review [26] detailing work conducted in this endeavour until 2010. There are a few notable follow-up studies published after this review. Using a dataset of 123 FDA approved drugs, Avgy-David and Senderowitz [27] showed that retaining 70% of conformers ranked by the energy difference between the Boltzmann average and the local minimum conformation retrieved a bioactive conformation for 80% of the ligands. On a larger dataset of 260 ligands, Habgood [28] showed that using a pluralistic approach compiling the top-ranked conformer from ascending potential energy and ascending solvation energy ranking leads to modest improvements of early enrichment of bioactive-like conformations compared to using the top-ranked conformers of a single ranking method. Therefore, descriptors such as the SASA or the RGyr, and the (strain) energy showed limited discriminative power of bioactive-like conformations among conformer ensembles, and further research is needed to find more information-rich descriptors.

Turning to the analysis of chemistry data, deep learning can be harnessed to extract information from conformations for fast and accurate property, energy, or force prediction, using machine learning potentials [29]. Deep learning has recently gained popularity in chemistry applications such as molecule generation [30, 31], 3D generation models [30, 32], molecular property prediction [33], conformation generation [34, 35] molecular docking [36, 37], binding affinity prediction [38, 39] or protein/complex structure prediction [40, 41]. As one particular implementation of deep learning models, atomistic neural networks (AtNN) process individual atom features along with encoding of their coordinates to obtain a single contribution per atom, that are summed to obtain a conformation-level output prediction. Initial AtNNs like ANI [42] or AIMNet [43] use atom-wise environment vectors with radial and angular features, produced using a modified version of the Behler and Parrinello symmetry functions [44], as input in atom-type specific neural networks to obtain energy prediction within chemical accuracy. Gilmer et al. [45] use a message passing neural network where raw interatomic distances are encoded in the edge feature (real or virtual bonds), to predict 13 QM9-computed properties [46] within chemical accuracy (as described in the work of Faber et al. [47]).

**Fig. 1:**
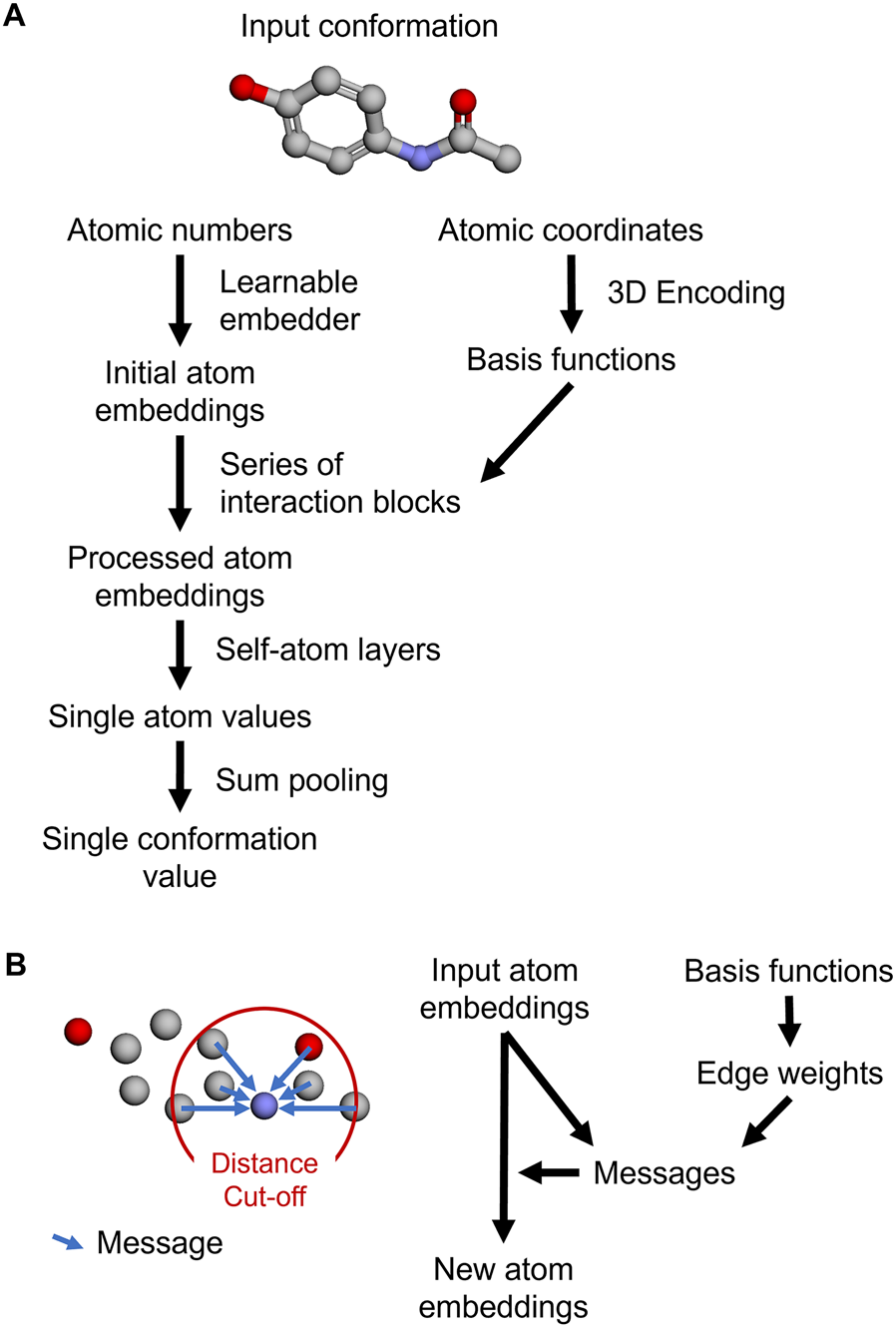
Schematic representation of AtNNs architecture. **A** AtNNs take as input a conformation in the form of atomic numbers with corresponding atomic coordinates to output a single value for the conformation. **B** Architecture of an interaction block. A new atom embedding is produced by using an input atom embedding and basis functions (encoding the 3D structure) through a message passing paradigm performing convolutions between atoms in a neighbourhood subgraph defined by a distance cut-off

AtNNs developed later followed the interaction block paradigm, pictured in Fig. 1, where the message passing is performed between raw atomic embeddings encoding at least the atomic number, convolving messages between neighbouring atoms (up to a certain cut-off) using basis function representations of 3D information such as distances, valence angles, or torsion angles; increasing amount of 3D information incorporated is often referred to as expressiveness of the graph neural network [48]. The mean absolute error of QM9 properties prediction using ComENet [49], that encodes distances along with valence angles and torsion angles, is on average half the one using the older SchNet model only encoding distances [50] (e.g., the mean absolute error of the HOMO-LUMO gap is 63 meV for SchNet and 32 meV for ComENet). Therefore, the recent more expressive AtNNs are state-of-the-art models for processing molecular conformations to predict their properties.

To our knowledge, AtNNs have not been used for bioactive conformation biasing, i.e., to extract specific information that discerns bioactive-like conformations from non-bioactive conformations of the same ligand. In this work, we trained AtNNs on generated conformers of a curated subset of PDBbind [51] ligands to predict the overlayed ARMSD of a given conformation to its closest known bioactive conformation. AtNN predictions were then used to rank an initial pool of conformers, with the aim of selecting a smaller conformer ensemble with an enrichment of bioactive-like conformations, to be further input in a rigid-ligand docking algorithm. Compared to the latest study on bioactive conformation biasing [28], the size of the PDBbind dataset after curation is a hundred times larger, allowing re-evaluation of previously identified descriptor (e.g., SASA or RGyr) and energy ranking methods on this larger dataset.

## Methods

### PDBbind data processing

Data on bioactive conformations were obtained from PDBbind [51] refined and general sets v2020, which is the largest dataset of protein-ligand complexes extracted from the Protein Data Bank (PDB) with corresponding binding affinity values. PDBbind metadata was extracted from the downloaded tab-separated index files. The starting PDBbind dataset contains 19,443 complexes for 12,921 unique ligands.

**Fig. 2:**
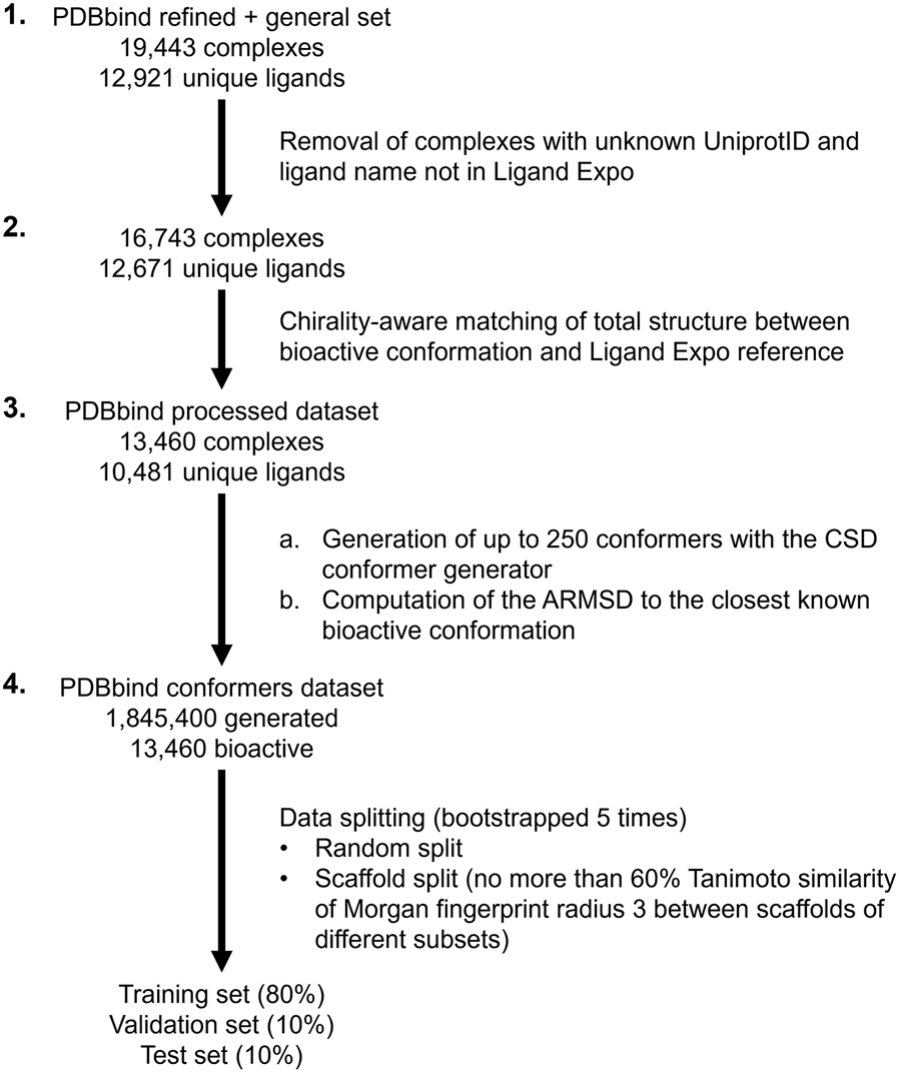
PDBbind data processing and splitting for modelling. Step **1**. The PDBbind dataset is limited to complexes with known Uniprot ID of proteins and ligand names in LigandExpo. Step **2**. Only ligands that matches the LigandExpo reference (chirality included) are kept. Step **3**. Up to 250 conformers for each unique ligand are generated (**a**), leading to a dataset of conformers. The ARMSD to the closest bioactive conformation is computed for each conformer (**b**). Step **4**. The dataset containing bioactive and generated conformers is split using a random or scaffold splitting

To restrict the current work to complexes with known metadata, the first step (step 1. in Fig. 2) was to remove PDBbind entries having no Uniprot accession number (the “Uniprot ID” field is filled with “------” for 354 complexes) and no ligand name (the field “ligand name” is “()” for 2 complexes). A ligand name cleaning was then applied, removing the extra parenthesis surrounding the 3-letter codes and applying some manual corrections listed in Additional file 1 to fit up-to-date ligand names in the PDB. PDBbind instances that didn’t have a 3-letter ligand name present in Ligand Expo [52], representing mostly polymers, oligosaccharides, or outdated 3-letter codes, were removed. This step left 16,743 complexes with 12,671 unique ligands from the original dataset.

To check if the ligand structures determined in the PDB corresponds to their reference structure in LigandExpo, the second step (step 2 in Fig. 2) was a chirality-aware structure matching between the two molecules as follows. The mapping between 3-letter code ligand names in the PDB to corresponding reference stereo-specific CACTVS SMILES structures was obtained from the LigandExpo website. SMILES were parsed using RDKit [53] v.2020.09.1.0 with Python 3.7.10 using the *MolFromSmiles* function. Bioactive conformations were extracted primarily from mol2 files using the *MolFromMol2File* function in RDKit. For the 2054 ligands for which the mol2 file could not be parsed (e.g., error in atom typing, or invalid valency), the sdf file was used instead using the *SDMolSupplier* function in RDKit. If the sdf file failed to be parsed as well, this conformation was not used. Hydrogen positions were not read from the mol2 and sdf files. The *AssignStereochemistryFrom3D* function in RDKit was used on molecules extracted from sdf files because they did not embed chirality, as opposed to mol2 files which has chirality embedded. Then, PDBbind and Ligand Expo molecules were standardised using the molvs package [54] 0.1.1 and neutralised using the *neutralize_mol* python function given in the RDKit Cookbook [55]. The two molecules were input to a chirality-aware chemical structure match in RDKit using the *GetSubstructMatch* function. If the matching failed, the *AssignBondOrdersFromTemplate* RDKit function was used since bond orders in PDBbind might be different to the one in Ligand Expo (as protonation states are recomputed in PDBbind), and matching was retried. After this matching step, we reached 13,460 conformations for 10,481 unique ligands where structures including chirality from the PDB matched that from LigandExpo.

To produce a diversity of energetically plausible conformers to train the model on, the third step (step 3a in Fig. 2) was to generate up to 250 conformers using the CSD Conformer Generator [10] for each unique ligand using the CSD Python API [56] v.3.0.9 (hydrogens are added during generation). 250 conformers were generated for 6478 unique ligands (62%) and less than 250 conformers were generated for the remaining low flexibility ligands, and hence a total of 1,845,400 conformations (the distribution of the number of generated conformers is shown in Additional file 1: Fig. S1E).

To measure how similar generated conformers of ligands are to known bioactive conformations, the fourth and final step (step 3b in Fig. 2) was to compute the ARMSD of each conformer to each bioactive conformation of its corresponding ligand, referred to as ARMSD_bio_. The ARMSD were computed using the CSD Python API *rmsd* function. We found at least one similar generated conformer using a 1 Å ARMSD threshold for 10,817 bioactive conformations (comprising 8513 unique ligands), representing an 80% retrieval (81% of unique ligands). This retrieval is lower than the 92% reported by Cole et al. [10] when evaluating the CSD conformer generator; however, the latter was done on the smaller Platinum dataset that contained ligands with an improved quality of the atom coordinates fitting to experimental electron density.

### Dataset splitting

The dataset was split into training, validation, and test subsets with ratios of number of unique ligands of 80%, 10% and 10%, respectively, using different splitting strategies (step 4 in Fig. 2). The random split corresponds to a random repartition of the unique ligands between the training, validation, and test set. The list of unique ligands is shuffled, then the first 80% is assigned to the training set, the next 10% to the validation set and the remaining 10% to the test set. The scaffold split was done by first computing the Bemis-Murcko scaffold of each ligand using the *GetScaffoldForMol* in RDKit, then computing the Morgan fingerprints radius 3 (from the family of Circular Fingerprints [57], similar to ECFP6) of the scaffold with RDKit, and clustering each ligand based on the scaffold fingerprint such that the minimal Tanimoto similarity within a cluster is 50% using the *fcluster* function in scipy [58]. Then the training subset was filled with randomly chosen clusters until the number of conformations covered reaches 80%, the validation subset was filled with clusters up to another 10% of conformations, and the test subset was filled with the remaining clusters. Each split was done 5 times to account for subset variability.

A ligand can have multiple bioactive conformations (one conformation per complex with a protein, to the same or different proteins, as shown in the ligand-target distribution analysis in Additional file 1: Fig. S1), and therefore a generated conformer for the ligand will have one ARMSD per corresponding bioactive conformation. As it is counter-intuitive for modelling to have multiple output ARMSD values for a unique input conformer, we decided to only use the lowest ARMSD_bio_ (i.e., the ARMSD to the closest bioactive conformation) for further analysis.

### Model architecture and training

AtNNs take an atomic point cloud as input in the form of atomic numbers and positions (bond types are not considered), as shown in Fig. 1A. The atomic numbers are in this network type generally converted to vectors using a learnable embedding (i.e., each atomic number is mapped to an initial vector that is learned by the model) and the atomic positions are used to compute interatomic distances, polar coordinates (encoding angles), and torsion angles. The atomic geometries are encoded with basis functions (inspired from physics) as edge features: for instance, SchNet [50] encodes distances using radial basis functions, DimeNet++ [59] encodes distances and polar coordinates respectively using Radial Bessel basis functions and 2D spherical Fourier-Bessel basis functions using spherical harmonics, while ComENet [49] encodes distances along with torsion angles, and polar coordinates by using two distinct sets of spherical Bessel basis functions using spherical harmonics. One neighbourhood subgraph per atom is built based on a distance cut-off. Interactions blocks use input atom embeddings and basis functions to produce new atom embeddings, pictured in Fig. 1B. Within an interaction block, the basis functions are processed to edge weights that will be combined with the input atom embedding using a message passing paradigm, performing convolutions between the atoms in the neighbourhood subgraph to update each atom embedding. After going through a series of interaction blocks, each processed atom embedding is then input in a final series of feed-forward neural networks to obtain a single output value per atom, that are summed to obtain a single output value for the conformation.

In this work, three different AtNNs were used to extract information from a 3D conformation to obtain a single output, namely SchNet [50], DimeNet++ [59] and ComENet [49] and for details of each method please see the original works cited. Pytorch v.1.8.0 [60] was used as the neural network library, using the Torch Geometric v.2.0.2 [61] implementation of the SchNet and DimeNet++ model and DIG v.1.0.0 [62] implementation of the ComENet model with default parameters (listed in “Default parameters for atomistic neural networks” section in Additional file 1).

The heavy atoms of the conformation were given as input of each AtNN considered, and the output of each AtNN model was trained to predict the ARMSD_bio_ using a mean-squared error loss (MSELoss in Pytorch) with the default Pytorch Adam optimizer [63] with a learning rate of 10^−5^ for SchNet and 10^−4^ for DimeNet++ and ComENet, after initial learning rate tuning to reach monotonically decreasing validation loss throughout training. Early stopping with a patience of 5 on the validation loss was setup to stop the model once the validation loss has stopped decreasing, keeping the model with the lowest validation loss. One instance of each AtNN per split was trained, resulting in 15 models per split type, totalling 30 models per AtNN. The average epoch duration was 6 min for SchNet, and 8 min each for DimeNet++ and ComENet using a computer running Ubuntu 20.04 with an AMD Ryzen 9 5900x CPU, one Nvidia RTX 3080 GPU (using CUDA) and 32 GB RAM, with an average of 20 epochs per model for the random splits, and 15 epochs for the scaffold splits.

### ARMSD regression evaluation

The regression performance of the model in retrieving the real ARMSD_bio_ value for input generated conformers of each test set was assessed with the root-mean-square error (RMSE) metric (using the *MSELoss* and *sqrt* functions in Pytorch), and the coefficient of determination R^2^ (using the *R2Score* in torchmetrics [64] v0.9.1) between all predicted and real ARMSD_bio_ values.

### Evaluation of the ranking of generated conformers

The primary objective of the current work was to test the ability of AtNN models to retrieve bioactive-like conformations among the top ranked conformers and reduce the rate of non-bioactive conformations. Conformers having an ARMSD_bio_ lower than 1 Å were labelled as bioactive-like conformations, while conformers having an ARMSD_bio_ higher than 2.5 Å were labelled as non-bioactive conformations, to stay consistent with the classification established by Musafia and Senderowitz [17]. For each molecule having at least one bioactive-like conformation and not having only bioactive-like conformations (as there would be no gain in ranking conformers in this case), generated conformers were ranked according to their AtNN predicted ARMSD_bio_. The ranks were scaled (i.e., divided by the number of generated conformers for the molecule) to range between 0 and 1, the rank of the first bioactive-like conformation and first non-bioactive conformation were stored, and the median rank per split was computed. Early enrichment of bioactive-like conformations and non-bioactive conformations were assessed using the Boltzmann-Enhanced Discrimination of Receiver Operating Characteristic (BEDROC) [65], which is a weighted version of the ROC metric, ranging between 0 and 1 to account for variable ratio of bioactive-like conformers per molecule, and giving higher values to conformer rankings having bioactive-like conformations in earlier ranks. The BEDROC $\alpha$ parameter was set to 20, according to Truchon et al. [65], where the presence of labelled conformations in the 8% top-ranked will contribute to 80% of the score. The BEDROC of bioactive-like conformations will from here on be referred to as BEDROC_bio-like_ while the BEDROC of non-bioactive conformations will be referred to as BEDROC_non-bio_.

### Generated conformers ranking baselines

#### Bioactivity-unaware baselines

Five bioactivity-unaware baselines (i.e., methods that do not relying on knowledge of the bioactive conformation) have been evaluated in their abilities to enrich bioactive-like conformation in early ranks. The first ranking baseline was to rank conformers using a random number from a standard normal distribution for each conformer using the Numpy [66] *random.randn* function, in ascending order, and referred to as “Random order”. Some conformer generators have (pseudo-)random generation order, and this baseline can mimic this scenario. This baseline evaluates how often we observe an early enrichment of bioactive-like conformations or impoverishment of non-bioactive conformations by chance.

The second ranking baseline was to keep the original CSD conformer generator order, according to the likelihood of the torsion angle based on profiles observed in the Cambridge Structural Database (CSD), referred to as “CSD Probability”. Cole et al. [10] showed on the diverse Platinum dataset that this conformer generator is able to retrieve a bioactive conformation for 90% of molecules using all conformers, 70% using the first 10% of conformers and 40% using the first generated conformation, using an ensemble size of 250 conformers, and therefore represents a strong baseline.

The third baseline was the Sage energy baseline, which is a ranking of conformers with ascending potential energy, as it was shown that in most of the cases, bioactive conformations have low strain energies to a local minima [21]. Single point energies were computed for each conformer (hydrogens were added using the *AddHs* in RDKit in case they were not embedded in the structure) using the Sage 2.1.0 force field [67] implemented in the OpenFF 0.14.5 Python toolkit [68].

The fourth baseline was the SASA baseline, that ranks conformers by descending SASA value, as it was shown that bioactive-like conformations tend to have higher SASA compared to other conformers [24, 26]. The SASA of each conformer was computed using the *rdFreeSasa.CalcSASA* function in RDKit.

The fifth baseline was the RGyr baseline, that ranks conformers by descending RGyr, as it was shown that bioactive-like conformations tend to be more elongated compared to other conformers [20, 24]. The RGyr of each conformer was computed using the *Descriptors3D.RadiusOfGyration* function in RDKit.

#### Bioactivity-based baseline

A sixth and last baseline depending on a reference dataset, in our case the training set, was designed, as opposed to the bioactivity-unaware approaches described in the previous subsection. For a given test molecule, the TFD2SimRefMCS baseline first identifies the closest molecule in the reference set using the Tanimoto similarity of Morgan fingerprints, then computes the Maximum Common Substructure (MCS) between the two molecules (using the rdFMCS module [69] in RDKit), calculates the Torsion Fingerprint Deviation (TFD) [70] using torsion angles involved in the MCS between each test molecule conformer and the bioactive conformation of the reference, and finally rank conformers according to the calculated TFD. Morgan fingerprints with radius 3 and including chirality, MCS with chirality matching and default TFD computations were performed in RDKit. For PDBbind molecules, the minimum TFD (across conformers) to reference MCS is lower than 0.25 for 80% of the molecules, as shown in Additional file 1: Fig. S2, indicating a low deviation to reference that can be harnessed to select bioactive-like conformations (which is also used in the CORES [71] method).

### Protein class dependent performance calculation

In order to analyse performance as a function of the protein target class, the ChEMBL database [72] version 29 was downloaded in SQLite format and loaded into Python with pandas v.1.2.5 [73] to extract the first level protein classification information for as many Uniprot ID in PDBbind as possible. The SQL query to extract data is given in Additional file 1. A protein class was found for 9428 complexes out of the 13,460 complexes present in the processed PDBbind dataset.

Since enzymes were the most represented protein class with 77% of complexes (7322 complexes), we additionally loaded the Enzyme classification data using the Enzyme Commission (EC) numbers from Expasy [74] where the available EC data was parsed in a table linking each Uniprot ID to the EC number. We obtained EC data for 8,800 complexes out of the 13,460 in the processed PDBbind dataset. Ranking performances between classes of the third EC level (out of four) was compared.

### Rigid-ligand docking seed selection

**Fig. 3:**
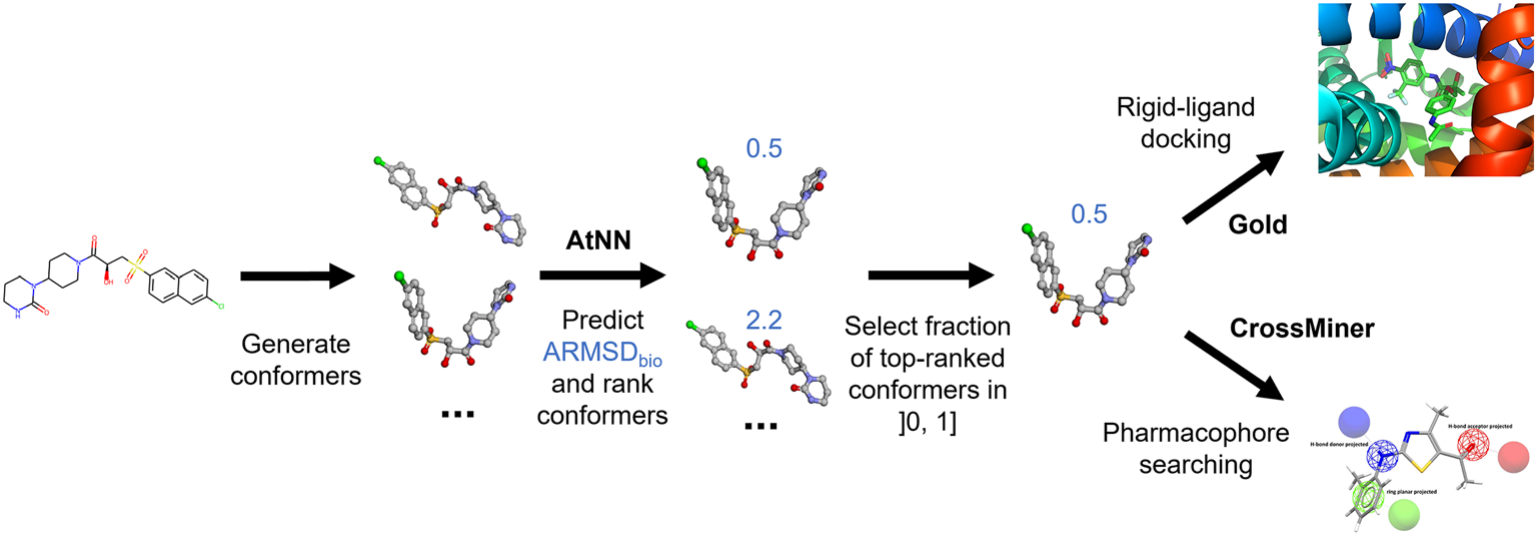
Rigid-ligand docking and pharmacophore searching workflow for one ligand, using the AtNN ranking method (blue numbers are predicted ARMSD_bio_ values). Up to 250 conformers have been first generated using the CSD conformer generator, then an AtNN is used to predict the ARMSD_bio_, that will be used to rank conformations and select only a fraction of top ranked conformers as rigid-docking seed or to be tested using a pharmacophore query. The AtNN ranking approach was compared to baselines

To evaluate real-world relevance of the methods developed here, the model capabilities of selecting bioactive-like conformations for rigid-ligand docking were assessed, with the objective of retrieving poses similar to the binding pose using a limited number of input conformations. For molecules having at least one bioactive-like conformation, AtNNs (or baselines) were used to rank conformers, and the highest-ranking fraction of 1%, 5%, 20% and 100% of all conformers were rigidly docked on the cognate protein, as shown in the workflow in Fig. 3. Proteins were prepared using the same sequence of functions with the CSD Python API, namely the *add_hydrogens*, *remove_ligand*, *remove_all_metals* and *remove_all_waters* functions. Rigid-ligand docking (*fix_ligand_rotatable_bonds* set to “all”) was performed with GOLD [75] in the CSD Python API, where binding sites were defined as protein atoms at a maximal distance of 6 Å from any cognate ligand atom, ten diverse poses were produced per conformer using the PLP scoring function, and the top-scoring pose per conformer was selected for downstream evaluation, i.e., a maximum of 250 poses per molecule for the 100% fraction.

For each fraction of selected conformers, the proportion of molecules having a correct pose with an ARMSD within 2 Å to the native pose was identified, referred to as successful docking rate, when taking the top-scoring pose, or when taking the pose with the closest ARMSD to the native pose (to mimic the case where poses closest to the native pose are given the best score). It is worth noting that the ARMSD threshold to label bioactive-like conformation (1 Å) is lower than the one generally used to discriminate successful docking poses (2 Å). The reason for this choice is that the former is an overlayed ARMSD (conformations are first aligned) while the latter is not overlayed (two poses are compared within the same frame, e.g., a binding pocket), leading to a larger number of degrees of freedom that makes it harder to retrieve the exact pose, hence the larger threshold chosen here for successful pose acceptance.

### Flexible ligand docking baseline

As opposed to rigid-ligand docking that only samples the position of the ligand in the pocket, flexible-ligand docking also samples values of torsion angles centred on rotatable bonds of the ligand, leading to different performances depending on the protein target as shown in an earlier study [76]. For each molecule tested in rigid-ligand docking, the first generated conformer was used as seed, and ten poses were produced. Parameters in GOLD were the same as the one used in rigid-ligand docking except for the rotatable bonds that were not fixed (*fix_ligand_rotatable_bonds* set to None).

### Pharmacophore searching

In addition to the rigid-ligand docking for structure-based virtual screening, the early ranking of bioactive-like conformers of the models and baselines was tested in a pharmacophore searching procedure to emulate ligand-based screening, as shown in Fig. 3. For each test set, the bioactive conformation of each molecule in the test set was used to produce a pharmacophore query using CrossMiner [77] through the CSD Python API: all pharmacophoric features among “donor projected”, “acceptor projected” and “ring planar projected” were computed for the conformation, and a random subset of up to 5 features were chosen to represent a pharmacophore that fits the bioactive conformation. The generated conformers of the corresponding molecule were screened against the pharmacophore query. The molecules having no generated conformer matching the pharmacophore were removed from the analysis, removing around half of the molecules in each test set, because either no bioactive-like conformation was present in the generated conformer set, or none of the bioactive-like conformations matched the pharmacophore (i.e., the pharmacophore search only compares the matching between features of the generated conformer and the query, while the ARMSD aggregates position difference between all heavy atoms). For fractions of the highest-ranking conformers of 1%, 5%, 20% and 100%, the fraction of molecule matching the pharmacophore query, referred to as hit rate, was computed (100% fraction takes all conformers, therefore the hit rate is guaranteed to be 100%).

## Results and discussion

### AtNN conformer ranking shows early enrichment of bioactive-like conformations on a par with the slower bioactivity-based baseline

**Fig. 4:**
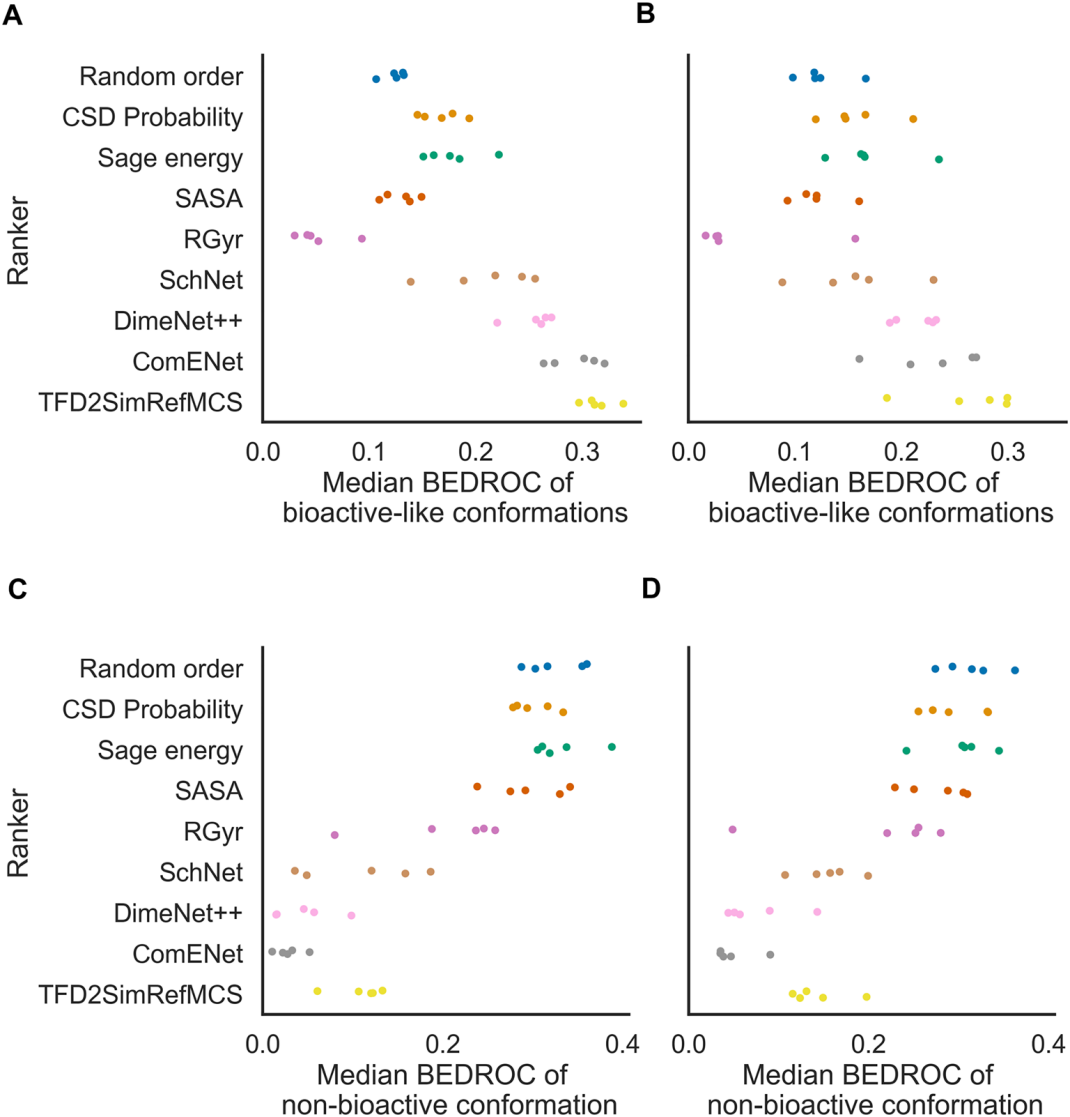
Median BEDROC of bioactive-like (**A**, **B**) and non-bioactive conformations (**C**, **D**) for all molecules of the test sets, for the random (**A**, **C**) and scaffold splits (**B**, **D**). Each point represents a split. AtNNs and TFD2SimRefMCS show higher median BEDROC of bioactive-like conformations than bioactivity-unaware baselines indicating better early enrichment of desirable conformations. They also show lower median BEDROC of non-bioactive conformations, indicating higher impoverishment (lower early enrichment) of undesirable conformations

We first analysed whether ARMSD_bio_ predicted by AtNNs could be used to rank generated conformers with the objective of retrieving a higher rate of bioactive-like conformations (ARMSD_bio_ ≤ 1 Å) and a lower rate of non-bioactive conformations (ARMSD_bio_ > 2.5 Å) in the early ranks. On random and scaffold splits of a curated subset of PDBbind, we trained AtNNs with increasing levels of expressiveness (i.e., completeness of embedded 3D information), namely SchNet, DimeNet++ and ComENet, on training sets to predict the ARMSD_bio_ then used the ARMSD_bio_ predictions in ascending order to rank generated conformers of corresponding test set molecules (regression performances are shown in “Regression results” section in Additional file 1: Fig. S3 and Table S1). We compared the AtNNs performance to five bioactivity-unaware baselines, that are randomly ordering conformers (Random order), initial conformer generator order based on CSD torsion angle probability (CSD Probability), Sage force field ascending energy ranking (Sage energy), decreasing solvent accessible surface area (SASA) ranking, and decreasing radius of gyration (RGyr) ranking. We also designed a bioactivity-based baseline, that ranks conformers by the Torsion Fingerprint Deviation (TFD) of the Maximum Common Substructure (MCS) to the bioactive conformation of the closest molecule in the training set (TFD2SimRefMCS). The early enrichment of bioactive-like conformations for each method in the random and scaffold splits is shown in Fig. 4A; Table 1. On the random split test sets, the median BEDROC_bio-like_ is 0.12 ± 0.01 for the Random order baseline, on a par with the SASA baseline with a median BEDROC_bio-like_ of 0.13 ± 0.02, while the early enrichment of bioactive-like conformation is lower for the RGyr baseline, with a median BEDROC_bio-like_ of 0.05 ± 0.02, which is an opposite result to what was observed in previous studies [20, 24] on much smaller datasets (i.e., less than 100 ligands) that showed that bioactive-like conformations had higher SASA and RGyr. On the other hand, the early enrichment of bioactive-like conformations is better for the CSD Probability baseline with a median BEDROC_bio-like_ of 0.17 ± 0.02, and for the Sage energy baseline with 0.18 ± 0.03. The TFD2SimRefMCS baseline leads to a median BEDROC_bio-like_ of 0.31 ± 0.02, showing better early enrichment of bioactive-like conformations for this bioactive-based method compared to all bioactivity-unaware baselines. While ranking with SchNet predictions, the least expressive AtNN, leads to a lower median BEDROC_bio-like_ of 0.21 ± 0.05, the most expressive ComENet model leads to a median BEDROC_bio-like_ of 0.29 ± 0.02, being the only AtNN to be on a par with the TFD2SimRefMCS baseline. Hence, more expressive AtNNs leads to better early enrichment of bioactive-like conformations than bioactivity-unaware baselines, and with similar performance to the bioactivity-based baseline.

**Table 1:** Median BEDROC_bio-like_ on test sets (mean ± standard deviation)

Ranker	Random split	Scaffold split
Random order	0.12 ± 0.01	0.13 ± 0.03
CSD probability	0.17 ± 0.02	0.16 ± 0.03
Sage energy	0.18 ± 0.03	0.17 ± 0.04
SASA	0.13 ± 0.02	0.12 ± 0.02
RGyr	0.05 ± 0.02	0.05 ± 0.06
SchNet	0.21 ± 0.05	0.16 ± 0.05
DimeNet++	0.26 ± 0.02	0.21 ± 0.02
ComENet	0.29 ± 0.02	0.23 ± 0.05
TFD2SimRefMCS	0.31 ± 0.02	0.26 ± 0.05

We next analysed the early enrichment of all methods on the scaffold split test sets, as shown in Fig. 4B; Table 1. SchNet ranking showed a median BEDROC_bio-like_ of 0.16 ± 0.05, on a par with the best bioactivity-unaware baseline that was Sage energy with a median BEDROC_bio-like_ of 0.17 ± 0.04. On the other hand, DimeNet++ and ComENet outperformed the bioactivity-unaware baselines with a median BEDROC_bio-like_ of 0.21 ± 0.02 and 0.23 ± 0.05 respectively, and on a par with the TFD2SimRefMCS baseline with a median BEDROC_bio-like_ of 0.26 ± 0.05, but showing lower values than their random split counterparts. These results also display the higher variability between the different scaffold splits compared to the random splits, with for instance a standard deviation of 0.05 for ComENet on the scaffold splits versus 0.02 on the random splits. Therefore, we observe similar trends but with higher variability for the median BEDROC_bio-like_ for each ranking method, and lower median BEDROC_bio-like_ for AtNNs than for the random split test sets.

We also compared the computational cost of ranking between AtNNs and the baselines. After an initial training lasting two hours on average, ranking conformers of molecules in a test set using SchNet, DimeNet++ and ComENet takes on average 70 s, 100 s and 80 s wall clock time respectively using a Nvidia RTX 3080 with CUDA. The TFD2SimRefMCS baseline required a higher runtime with an average of 300 s, while the CSD Probability baseline does not require any additional processing, the Random order baseline takes one random shuffling iteration (requiring less than one microsecond of runtime), and the Sage energy requires around 500 s of wall clock time. Therefore, using the most expressive AtNN leads to an early enrichment of bioactive-like conformations comparable to using the bioactivity-based baseline at a lower processing cost.

### AtNN conformer ranking shows early impoverishment of non-bioactive conformations on a par with the bioactivity-based baseline

We next evaluated if the model could help removing non-bioactive conformations from the early ranks, with the objective to reduce the number of false positives from potentially wrong poses in rigid-ligand docking or avoid wrong hits in pharmacophore searching. This is referred to by Musafia and Senderowitz [17] as early ‘impoverishment’. To this end, we computed the median BEDROC of non-bioactive conformations, shown in Fig. 4C; Table 2. The Random order baseline shows a median BEDROC_non-bio_ of 0.32 ± 0.03 while the CSD Probability, Sage energy, and SASA baselines show similar median BEDROC_non-bio_ of 0.30 ± 0.02, 0.33 ± 0.03 and 0.30 ± 0.04 respectively, indicating similar impoverishment of non-bioactive conformations. It is worth noting that for the Random order baseline, the BEDROC_non-bio_ (0.32) is higher than the BEDROC_bio-like_ (0.12), this is due to the number of non-bioactive conformations being higher than the number of bioactive-like conformations for most molecules, and the parameter $\alpha$ of the BEDROC equal to 20, where the presence of labelled (i.e., bioactive-like, or non-bioactive) conformations in the 8% top-ranked contributes to 80% of the score. The RGyr baseline show lower median BEDROC_non-bio_ of 0.2 ± 0.07 compared the other bioactivity-unaware baselines, indicating a better early impoverishment of non-bioactive conformations. While the RGyr baseline was not better than the Random order baseline for early enrichment of bioactive-like conformation, it shows worse early impoverishment of non-bioactive conformations (i.e., having less more non-bioactive conformations in early ranks), suggesting that non-bioactive conformations have on average lower RGyr values in the tested molecules. SchNet, DimeNet++, ComENet and the TFD2SimRefMCS baseline showed median BEDROC_non-bio_ of 0.11 ± 0.07, 0.05 ± 0.03, 0.03 ± 0.02 and 0.10 ± 0.03 respectively, indicating an improved early impoverishment compared to bioactivity-unaware baselines. On the scaffold split test sets, we observe similar values for the bioactivity-unaware baselines while AtNNs and TFD2SimRefMCS show slightly higher median BEDROC_non-bio_, as shown in Fig. 4D; Table 2, indicating similar trends. Therefore, these results indicate that conformer ranking using AtNNs leads to early enrichment of bioactive-like conformations and impoverishment of non-bioactive conformations on a par with the slower TFD2SimRefMCS and better than bioactivity-unaware baselines.

**Table 2:** Median BEDROC_non-bio_ on test sets (mean ± standard deviation)

Ranker	Random split	Scaffold split
Random order	0.32 ± 0.03	0.31 ± 0.03
CSD probability	0.3 ± 0.02	0.3 ± 0.04
Sage energy	0.33 ± 0.03	0.3 ± 0.04
SASA	0.3 ± 0.04	0.28 ± 0.04
RGyr	0.2 ± 0.07	0.21 ± 0.09
SchNet	0.11 ± 0.07	0.15 ± 0.03
DimeNet++	0.05 ± 0.03	0.08 ± 0.04
ComENet	0.03 ± 0.02	0.05 ± 0.02
TFD2SimRefMCS	0.11 ± 0.03	0.14 ± 0.03

### For flexible ligands with a low number of bioactive-like conformations, bioactivity-based methods outperform bioactivity-unaware baselines for early enrichment of bioactive-like conformations

**Fig. 5:**
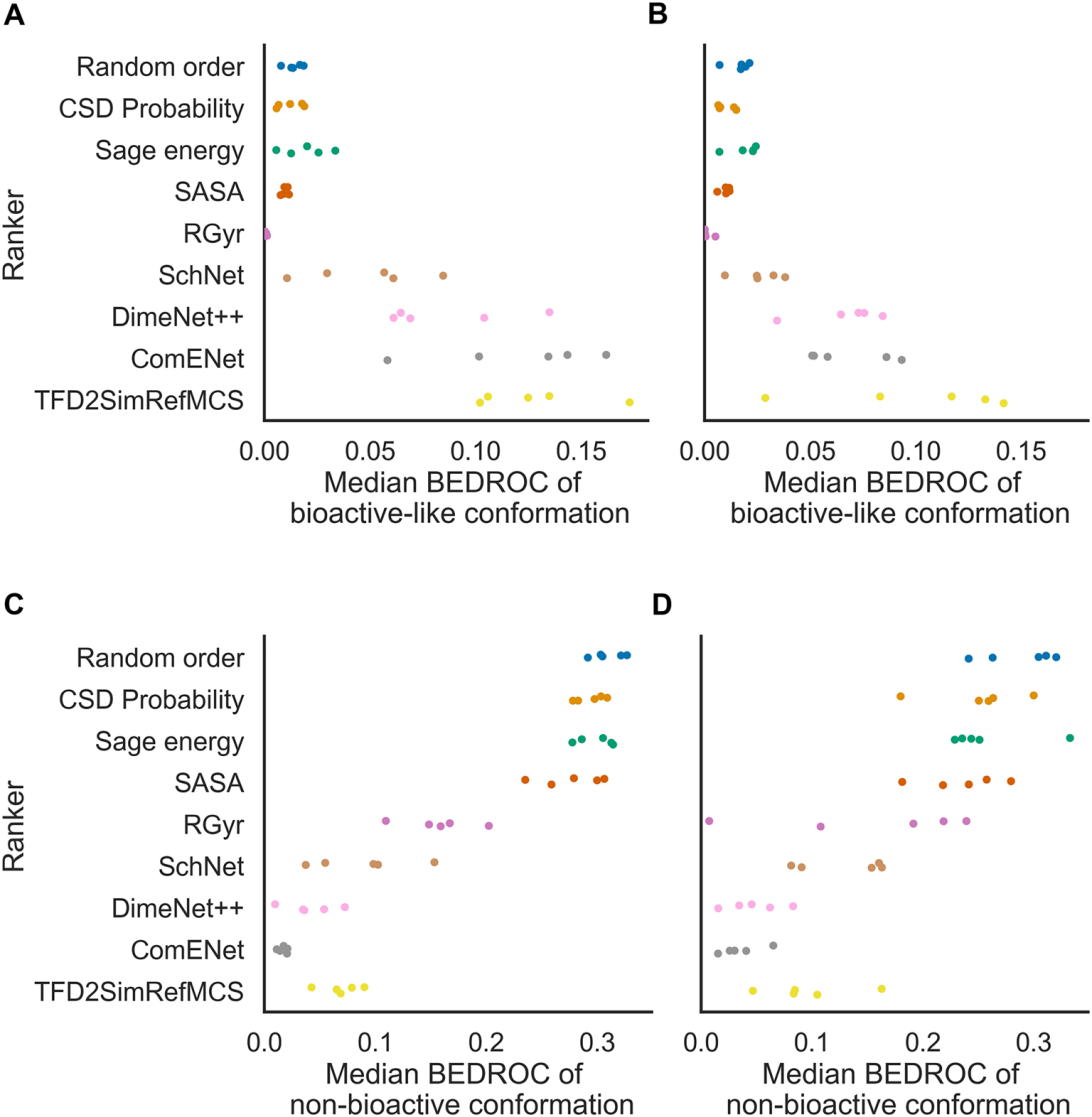
Median BEDROC of bioactive-like (**A**, **B**) and non-bioactive conformations (**C**, **D**) on harder test sets containing only molecules having 250 generated conformers and less than 5% bioactive-like conformations, for the random (**A**, **C**) and scaffold splits (**B**, **D**). Each point represents a split. AtNNs and TFD2SimRefMCS show higher median BEDROC of bioactive-like conformations than bioactivity-unaware baselines indicating better early enrichment of desirable conformations. They also show lower median BEDROC of non-bioactive conformations, indicating higher impoverishment (lower early enrichment) of undesirable conformations

Ranking performances evaluated on all ligands might be overestimated, as the enrichment of bioactive-like conformations might be biased by the number of generated conformers (i.e., higher for molecules having low flexibility) or the ratio of bioactive-like conformations (i.e., higher for molecules having a lot of bioactive conformations). We thus identified a restricted subset of molecules having 250 generated conformers with fewer than 5% of bioactive-like conformations. This represented a total of 1485 molecules, on average 214 per test set, representing around 29% of analysed ligands, for which it is harder to find a bioactive conformation by chance in early ranks. As opposed to the conformer ranking analysis that was performed on the complete test sets in the previous section, we evaluated the early enrichment of bioactive-like conformations on molecules from this ‘hard’ test set only. We found that the bioactivity-unaware baselines failed at enriching bioactive-like conformations in the early ranks, with the best baseline being the Sage energy baseline with a median BEDROC_bio-like_ of 0.02 ± 0.01, as shown in Fig. 5A; Table 3. On the other hand, DimeNet++, ComENet and the TFD2SimRefMCS baseline showed higher enrichment, with 0.09 ± 0.03, 0.12 ± 0.04 and 0.13 ± 0.02 respectively. For each ranking method, the median BEDROC_bio-like_ on these harder test sets are less than half the median BEDROC_bio-like_ on the full test sets. ComENet showed an average median BEDROC_bio-like_ of 0.30 versus the Sage energy showing 0.18 on the full test sets, representing a 1.6-fold increase, while there is a sixfold increase on this hard subset. On the scaffold split test sets, SchNet showed a median BEDROC_bio-like_ of 0.03 ± 0.01, that did not outperform the three bioactivity-unaware baselines like the Random order baseline with a median BEDROC_bio-like_ of 0.02 ± 0.01, as shown in Fig. 5B; Table 3. DimeNet++ and ComENet showed a similar median BEDROC_bio-like_ value with 0.07 ± 0.02 (threefold increase), outperforming bioactivity-unaware baselines, and on a par with the TFD2SimRefMCS baseline with 0.10 ± 0.05 (fivefold increase). Hence, AtNN and TFD2SimRefMCS ranking methods outperform the bioactive-unaware baselines for the early enrichment of bioactive-like conformations with a greater difference ratio on a more difficult subset, compared to the whole test set.

**Table 3:** Median BEDROC_bio-like_ on harder test sets (mean ± standard deviation)

Ranker	Random split	Scaffold split
Random order	0.01 ± 0.0	0.02 ± 0.01
CSD probability	0.01 ± 0.01	0.01 ± 0.0
Sage energy	0.02 ± 0.01	0.02 ± 0.01
SASA	0.01 ± 0.0	0.01 ± 0.0
RGyr	0.0 ± 0.0	0.0 ± 0.0
SchNet	0.05 ± 0.03	0.03 ± 0.01
DimeNet++	0.09 ± 0.03	0.07 ± 0.02
ComENet	0.12 ± 0.04	0.07 ± 0.02
TFD2SimRefMCS	0.13 ± 0.03	0.1 ± 0.05

We also investigated the ranking metrics of non-bioactive conformations on this hard subset. The ranges of values are shown in Fig. 5C, D; Table 4, and they are similar to those observed on the whole test set, in that bioactivity-unaware baselines showed median BEDROC_non-bio_ higher than 0.2, while it did not exceed 0.1 for AtNNs and TFD2SimRefMCS. Therefore, AtNN and TFD2SimRefMCS ranking methods also outperform the bioactivity-unaware baselines for the impoverishment of non-bioactive conformations in early ranks in the more difficult subset.

**Table 4:** Median BEDROC_non-bio_ on harder test sets (mean ± standard deviation)

Ranker	Random split	Scaffold split
Random order	0.31 ± 0.01	0.29 ± 0.03
CSD probability	0.29 ± 0.01	0.25 ± 0.04
Sage energy	0.3 ± 0.02	0.26 ± 0.04
SASA	0.28 ± 0.03	0.24 ± 0.04
RGyr	0.16 ± 0.03	0.15 ± 0.1
SchNet	0.09 ± 0.05	0.13 ± 0.04
DimeNet++	0.04 ± 0.02	0.05 ± 0.03
ComENet	0.02 ± 0.0	0.04 ± 0.02
TFD2SimRefMCS	0.07 ± 0.02	0.1 ± 0.04

### AtNN ranking performance advantage over bioactivity-unaware baselines is observed exclusively on protein classes overrepresented in PDBbind

We next assessed if ranking performance was dependent on the number of samples per protein class, as we observe an unequal distribution of complexes for ChEMBL protein classes, as shown in Additional file 1: Table S2. As the SASA and RGyr baselines did not show better early enrichment of bioactive-like conformations or early impoverishment of non-bioactive conformations than random, they were not considered in the following analysis. We grouped the ligands by corresponding protein class and computed the median BEDROC_bio-like_ and BEDROC_non-bio_ per split. On the random split test sets, for the enzyme protein class, being the most represented class (7322 out of 9902 labels), the median BEDROC_bio-like_ is 0.30 ± 0.02 for ComENet, 0.34 ± 0.01 for the TFD2SimRefMCS baseline and 0.18 ± 0.02 for the Sage energy baseline, showing that the AtNN and TFD2SimRefMCS baseline are consistently showing better early enrichment than the bioactivity-unaware baselines, as shown in Additional file 1: Fig. S4 and Table S2. For the other classes, the inter-split variability can exceed 0.05 BEDROC units, thus it is unsure whether the AtNNs and the TFD2SimRefMCS baseline are really outperforming the other baselines: on transcription factors, the TFD2SimRefMCS baseline shows a median BEDROC_bio-like_ of 0.26 ± 0.08, ComENet a median BEDROC_bio-like_ of 0.25 ± 0.12, while the Sage energy baseline shows a median BEDROC_bio-like_ of 0.18 ± 0.07. On the scaffold splits test sets, for the enzyme class, the best AtNN ComENet shows a median BEDROC_bio-like_ of 0.24 ± 0.04, higher than the best bioactivity-unaware baseline Sage energy with a median BEDROC_bio-like_ of 0.18 ± 0.03, and lower than the TFD2SimRefMCS baseline with a median BEDROC_bio-like_ of 0.29 ± 0.04. The high inter-split variability (higher than 0.05 BEDROC units) observed for the other protein classes does not allow to differentiate performances between all methods. For the early impoverishment of non-bioactive conformations, the median BEDROC_non-bio_ for enzymes is consistently over 0.25 for bioactivity-unaware baselines, while for bioactivity-based methods it is below 0.10 on the random splits and below 0.15 on the scaffold splits, and the high inter-split variability for other classes is also observed here. Therefore, the bioactivity-based methods only show improved early enrichment of bioactive-like conformations and impoverishment of non-bioactive conformations for enzymes.

We then focused on enzymes in more detail, separating the different classes using the third level of the EC classification. There is also an unequal distribution of samples in these enzyme classes, as shown by the histogram of the number of complexes per enzyme class in Additional file 1: Fig. S5. When ranking conformers, we found that AtNN and TFD2SimRefMCS baselines consistently outperform the bioactivity-unaware baselines on the median BEDROC_bio-like_ for the first, third and sixth most represented enzyme classes out of 134 classes, namely the 2.7.11 class of protein-serine/threonine kinases with a median BEDROC_bio-like_ of 0.31 ± 0.05 for ComENet versus 0.22 ± 0.04 for the Sage energy, the 3.4.21 class of the serine endopeptidases with a median BEDROC_bio-like_ of 0.39 ± 0.07 for ComENet versus 0.12 ± 0.06 for the Sage energy, and the 3.4.23 class of aspartic endopeptidases with a median BEDROC_bio-like_ of 0.45 ± 0.16 for ComENet versus 0.15 ± 0.07 for the Sage energy, as shown in Additional file 1: Fig. S6A, B and Table S3. For the other enzyme classes, there is no observed difference between the AtNN ranking and bioactivity-unaware baselines (e.g., for the fourth most represented enzyme class that is 4.2.1, comprising hydro-lyases), or there is a large inter-split variability, as shown for the eighth most represented class that is 2.1.1 (comprising methyltransferases), where the median BEDROC_bio-like_ ranges between 0 and 0.85 for SchNet ranking. Similarly, we analysed the early impoverishment of non-bioactive conformations using the median BEDROC_non-bio_. We observed that AtNN and TFD2SimRefMCS baselines consistently outperforms the bioactivity-unaware baselines for the three most-represented enzyme classes, with median BEDROC_non-bio_ on average under 0.2 for AtNN and TFD2SimRefMCS ranking, and median BEDROC_non-bio_ on average over 0.2 for the bioactivity-unaware baselines. These results indicate that the AtNNs and bioactivity-based baseline show better enrichment of bioactive-like conformations and impoverishment of non-bioactive conformations for the most-represented protein target classes in the training/reference data. Hence, if we have more ligands in the training set for an enzyme of interest, we are more likely to identify bioactive-like conformations of new ligands.

### AtNN ranking is more efficient on test molecules having a large MCS to training molecules

**Fig. 6:**
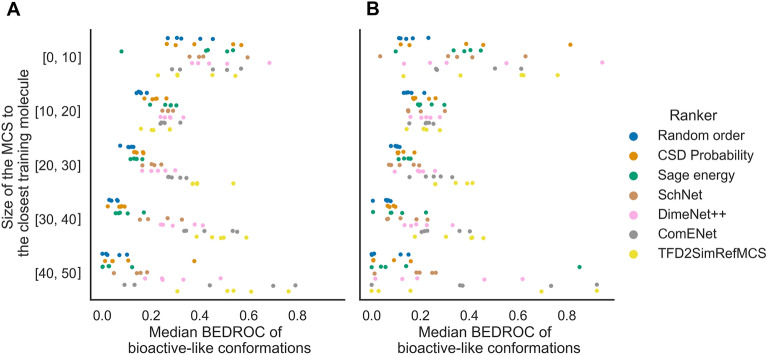
Median BEDROC of bioactive-like conformations depending on the size (number of heavy atoms) of the MCS to the closest training molecule, for the random (**A**) and scaffold (**B**) split test sets. Each point represents a split. For MCS sizes between 20 and 40 heavy atoms, AtNNs and TFD2RefSim rankers outperforms the bioactivity-unaware baselines

We next analysed whether the size of the MCS to the training set molecule with the highest Tanimoto similarity of Morgan fingerprint (with radius 3) was an indicator of good model performance. We grouped the MCS sizes by bins of 10 heavy atoms and show the distribution of median BEDROC_bio-like_ for each splitting strategy in Fig. 6; Table 5. We observe that for MCS sizes between 20 and 40 heavy atoms, the AtNNs and TFD2SimRefMCS ranking show better early enrichment than the bioactivity-unaware baselines on the random split, as shown in Fig. 6A: for the bin “[30, 40[”, ComENet ranking shows a median BEDROC_bio-like_ of 0.44 ± 0.10, the TFD2SimRefMCS baseline shows 0.48 ± 0.07 while the best bioactivity-unaware baseline Sage energy shows 0.09 ± 0.05. There is no early enrichment advantage of bioactivity-based methods over bioactivity-unaware methods when the MCS size is lower than 20 heavy atoms. Additionally, we compared the size of the MCS and the minimum TFD (as computed by the TFD2SimRefMCS ranker) for molecules in PDBbind depending on whether the closest reference molecule shared the same enzyme class or not. When the reference molecule has the same enzyme class, the size of the MCS was larger than when the reference molecule has a different enzyme class, with 24 ± 8 versus 16 ± 7 heavy atoms, indicating that ligands binding to the same enzyme class in PDBbind have similar substructures, as well as the median minimum TFD that was lower with 0.04 versus 0.12, as shown in Additional file 1: Fig. S2. We can hence conclude that the AtNN and TFD2SimRefMCS ranking methods showed better early enrichment of bioactive-like conformations than bioactivity-unaware baselines for ligands having a MCS larger than 20 heavy atoms to a training molecule.

**Table 5:** Median BEDROC_bio-like_ for different MCS size to training molecules (mean ± standard deviation)

Test set	MCS size binned	Random order	CSD probability	Sage energy	SchNet	DimeNet++	ComENet	TFD2SimRefMCS
Random	[0, 10[	0.35 ± 0.08	0.41 ± 0.14	0.4 ± 0.18	0.44 ± 0.11	0.48 ± 0.13	0.43 ± 0.12	0.37 ± 0.13
	[10, 20[	0.16 ± 0.02	0.22 ± 0.03	0.26 ± 0.04	0.27 ± 0.02	0.28 ± 0.04	0.26 ± 0.03	0.23 ± 0.05
	[20, 30[	0.11 ± 0.02	0.15 ± 0.02	0.14 ± 0.02	0.21 ± 0.03	0.23 ± 0.05	0.3 ± 0.03	0.41 ± 0.07
	[30, 40[	0.04 ± 0.02	0.07 ± 0.03	0.09 ± 0.05	0.23 ± 0.08	0.31 ± 0.08	0.44 ± 0.1	0.48 ± 0.07
	[40, 50[	0.04 ± 0.04	0.12 ± 0.15	0.04 ± 0.05	0.13 ± 0.06	0.3 ± 0.12	0.43 ± 0.32	0.55 ± 0.17
Scaffold	[0, 10[	0.15 ± 0.05	0.39 ± 0.28	0.33 ± 0.14	0.35 ± 0.21	0.44 ± 0.32	0.38 ± 0.17	0.5 ± 0.25
	[10, 20[	0.16 ± 0.03	0.2 ± 0.04	0.23 ± 0.05	0.19 ± 0.07	0.22 ± 0.04	0.22 ± 0.04	0.21 ± 0.05
	[20, 30[	0.1 ± 0.01	0.13 ± 0.03	0.14 ± 0.02	0.13 ± 0.06	0.19 ± 0.06	0.25 ± 0.07	0.33 ± 0.07
	[30, 40[	0.05 ± 0.03	0.08 ± 0.01	0.1 ± 0.08	0.13 ± 0.07	0.21 ± 0.08	0.28 ± 0.09	0.35 ± 0.11
	[40, 50[	0.06 ± 0.07	0.05 ± 0.07	0.22 ± 0.36	0.18 ± 0.1	0.28 ± 0.25	0.48 ± 0.36	0.36 ± 0.42

### Analysing ComENet early enrichment success

**Fig. 7:**
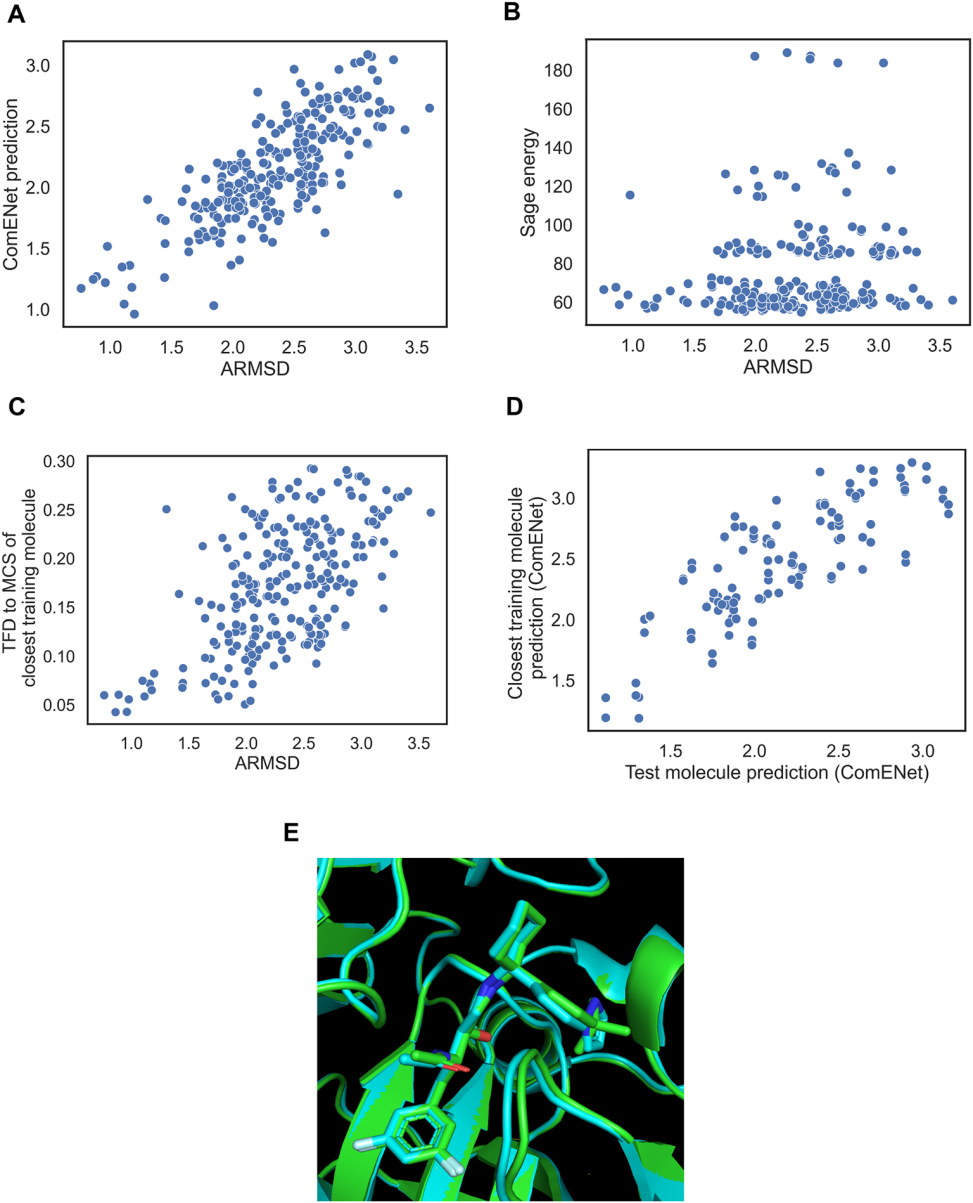
Inspection of a ComENet success case of early retrieval of bioactive-like conformations for the ILI ligand in the 3ivh complex. **A** Actual ARMSD_bio_ compared to ComENet prediction. **B** ARMSD_bio_ compared to the Sage energy. **C** ARMSD_bio_ compared to the TFD to the MCS of the closest molecule in training set. **D** Comparison between the ComENet predictions of the generated conformers of the test molecule and the predictions of generated conformers of the closest molecule based on matching conformers using torsion angles of their MCS. **E** Overlay between the bioactive pose of the ILI ligand in the 3ivh complex (green) and closest ligand “842” binding the same protein pocket in the 3n4l complex (cyan)

We next analysed several individual ranking examples for ligands of the most represented enzyme classes to try to understand cases where ComENet succeeds or fails at enriching bioactive-like conformations in early ranks. We started with the ranking of generated conformers of the ILI ligand from the 3ivh complex in the first random split test set. The bioactivity-unaware baselines failed in identifying bioactive-like conformations in early ranks, with BEDROC_bio-like_ ranging from 0.005 for the Random order baseline and the Sage energy baseline. ComENet outperformed the bioactivity-unaware baselines, with a BEDROC_bio-like_ of 0.68. The TFD2SimRefMCS baseline outperformed the AtNNs, with a BEDROC_bio-like_ of 0.85. Relating ARMSD_bio_ and the ComENet prediction, shown in Fig. 7A, it can be seen that ComENet predictions are positively correlated with the ARMSD_bio_, with a Pearson coefficient of 0.78, meaning that conformations with low ComENet prediction have on average lower ARMSD_bio_, increasing the chance of finding bioactive-like conformations at early ranks when sorting by ascending ComENet prediction.

We next analysed the Sage energies with regards to the ARMSD_bio_, shown in Fig. 7B. At best, there is a weak correlation between these two features (Pearson coefficient = 0.12, p-value = 0.05), meaning that the low-energy conformers of this ligand do not have a low ARMSD_bio_ (i.e., the bioactive conformation is strained). We then investigated the relationship between the ARMSD_bio_ and the TFD of the MCS to bioactive conformation of the closest molecule in the training set, which was the “842” ligand, sharing a large MCS representing more than half of each molecule with 30 heavy atoms. As shown in Fig. 7C, the two values are positively correlated, with a Pearson coefficient of 0.59, and showing that bioactive-like conformations have low TFD values, lower than 0.10. This means that the model identified conformers of the test molecule having similar 3D configuration of the MCS to the closest training molecule.

We next analysed whether the model captured any similarity between the 3D structure of the MCS. We matched similar generated conformations between the two molecules based on the torsion angles involved in the MCS, binning torsion angles by 10 degrees, and we predicted values using ComENet for each conformation in a matched pair, showed in Fig. 7D. The predicted values between pairs of conformations with similar MCS are positively correlated with a Pearson coefficient of 0.87. Hence, we can conclude that the model learned to give similar output values for molecules that share similar 3D configuration of their MCS.

To compare the bioactive conformations of the test molecule and closest training molecules, we superimposed the complexes with PDB codes 3ivh and 3n4l (where the “842” ligand is bound to the same protein), shown in Fig. 7E. We observed that the bioactive conformations have a similar binding pose for the MCS, which explains the correlation between predicted ARMSD_bio_ and how the model was able to retrieve bioactive-like conformations of the ILI ligand. These results suggest that the model is learning ARMSD_bio_ bias from specific substructure conformations that are present in the training set, helping the prediction for a substructure in the test set.

### Analysing ComENet early enrichment failures

**Fig. 8:**
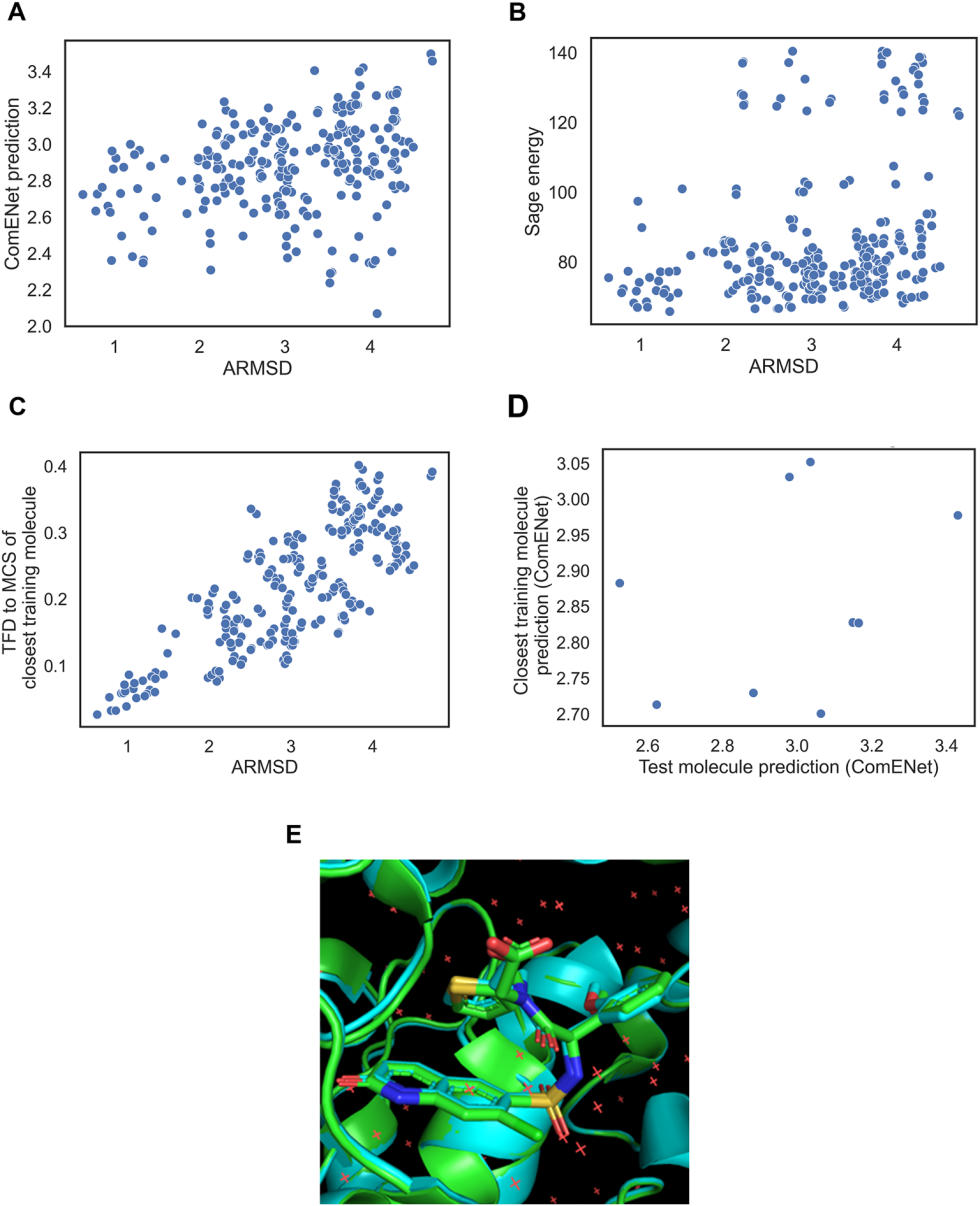
Inspection of a ComENet failure case of early retrieval of bioactive-like conformations for the JA4 ligand in the 6ma1 complex. **A** Actual ARMSD_bio_ compared to ComENet prediction. **B** ARMSD_bio_ compared to the Sage energy. **C** ARMSD_bio_ compared to the TFD to the MCS of the closest molecule in training set. **D** Comparison between the ComENet predictions of the generated conformers of the test molecule and the predictions of generated conformers of the closest molecule based on matching conformers using torsion angles of their MCS. **E** Overlay between the bioactive pose of the JA4 ligand in the 6ma1 complex (green) and closest ligand J9V binding the same protein pocket in the 6ma5 complex (cyan)

We next analysed cases where the model fails at early enrichment of bioactive-like conformations despite having a large MCS to the training set. For the ranking of the JA4 ligand in the 6ma1 complex, Sage energy ranking outperforms ComENet ranking for early enrichment with BEDROC_bio-like_ of 0.191 and 0.100 respectively. The TFD2SimRefMCS ranker was the best method with a BEDROC_bio-like_ of 0.897. The positive correlation of ARMSD_bio_ with the predicted ComENet values (Pearson coefficient = 0.29), as shown in Fig. 8A, is on a par with its correlation with the Sage energy (Pearson coefficient = 0.25), as shown in Fig. 8B, both lower than its correlation with TFD to MCS of closest reference molecule (Pearson coefficient = 0.78), as shown in Fig. 8C. The only difference between the test ligand and the closest ligand (J9V ligand seen in the 6ma5 complex) in the training set is the addition of a chlorine atom, as visualised on Fig. 8E. However, we retrieve no apparent correlation between the ComENet predictions when matching conformers with torsion angles in the MCS using bins of 20 degrees, shown in Fig. 8D. This result shows that a single atom difference between two chemical structures can lead to very different AtNN predictions. When looking at other test ligand predictions, we observe similar behaviour when training set molecules were similar except adding/removing halogen atoms (e.g., 4lwv ligand similar to 4lwu ligand, or 5t18 ligand similar to 5jrs ligand, data not shown). This might be due to the under-representation of halogen atoms in the molecules in PDBbind, making the neural network learning less from halogen atomic environment, and failing to produce accurate ARMSD_bio_ on the test ligand. Hence one case where model performance significantly deteriorates is when ranking molecules that differ from the training set structures by a halogen atom.

**Fig. 9:**
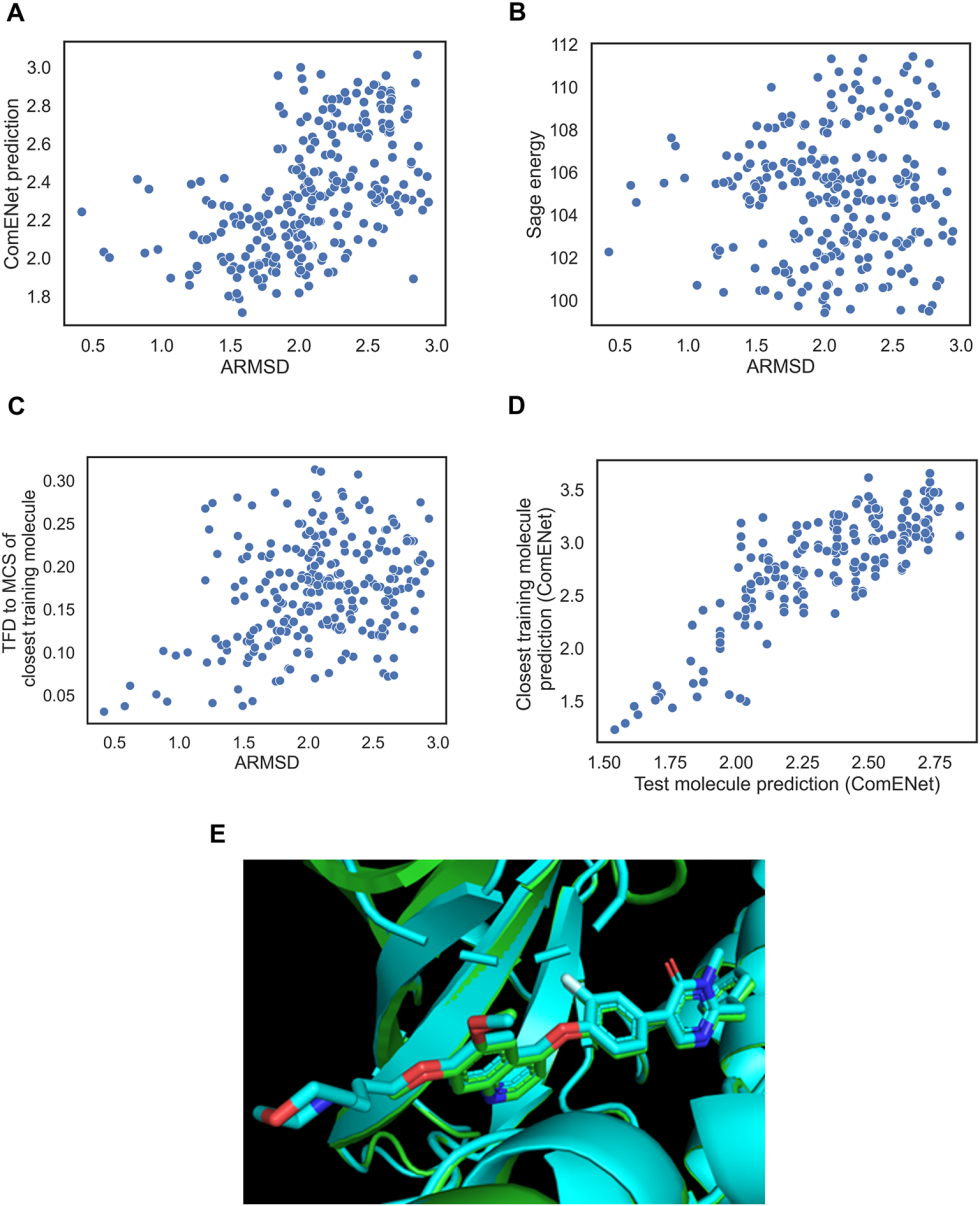
Inspection of a ComENet failure case of early retrieval of bioactive-like conformations for the MT3 ligand in the 3efj complex. **A** Actual ARMSD_bio_ compared to ComENet prediction. **B** ARMSD_bio_ compared to the Sage energy. **C** ARMSD_bio_ compared to the TFD to the MCS of the closest molecule in training set. **D** Comparison between the ComENet predictions of the generated conformers of the test molecule and the predictions of generated conformers of the closest molecule based on matching conformers using torsion angles of their MCS. **E** Overlay between the bioactive pose of the MT3 ligand in the 3efj complex (green) and closest ligand AM7 binding the same protein pocket in the 2rfn complex (cyan)

Another failure mode of the AtNN ranking are cases where the test set molecule is a substructure of a larger training molecule, such as the MT3 ligand in the 3efj complex being a substructure of the AM7 ligand from the 2rfn complex. Both ligands show the same binding pose of their MCS, but the training set ligand has an extra tail directed towards outside the binding pocket, as pictured in Fig. 9E. The AtNN learned to recognize the correct structure of the MCS as shown by the positive correlation between the ComENet prediction of the test molecule MCS and closest training molecule MCS when matching torsion angles (bins of 10°), with a Pearson coefficient of 0.74, as shown in Fig. 9D. However, the overall ComENet predicted values are not correct due to the addition of new atoms, as shown in Fig. 9A. Adding new atoms adds terms to the sum of atomic contributions (that results in the single value ARMSD_bio_ prediction) and modifies the atomic environment that each interaction block is processing to obtain atomic contributions. Hence, another case where the AtNN ranking does not outperform TFD2SimRefMCS baseline is when the tested ligand is a substructure of a larger training ligand. Therefore, even when the MCS between the test molecule and its closest training molecule is larger than 20 heavy atoms, the model is prone to error due to specific atom changes or for significant molecular size difference.

### Selecting the AtNN highest-ranked conformers for GOLD rigid-ligand re-docking of PDBbind complexes leads to higher successful docking rate than bioactivity-unaware baselines

**Fig. 10:**
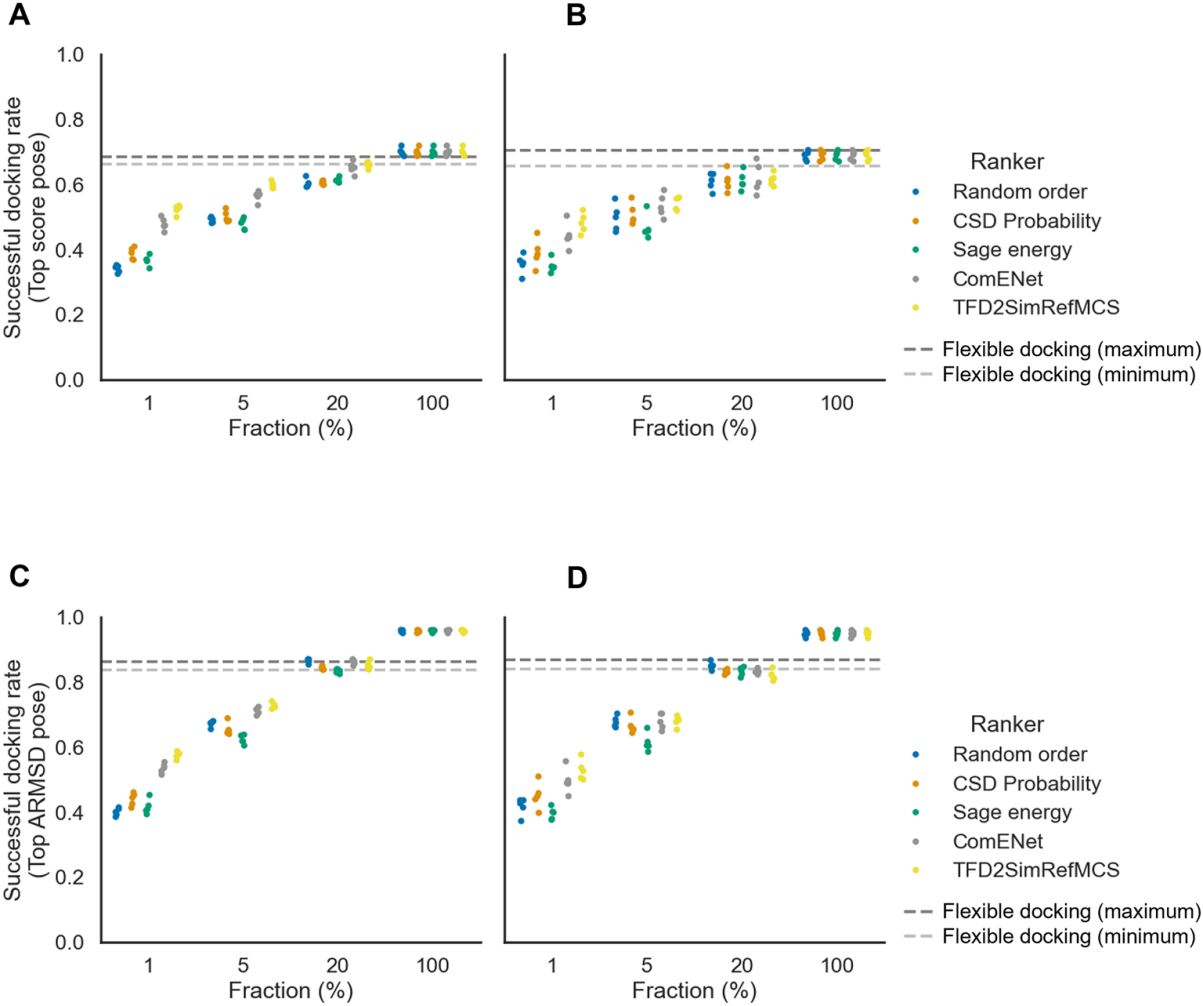
Successful docking rate of the highest score pose (**A**, **B**) or lowest ARMSD pose (**C**, **D**) for GOLD rigid-ligand redocking of PDBbind selecting various fractions of conformers per docked ligand using different rankers, for the random (**A**, **C**) and scaffold (**B**, **D**) splits test sets. Each point represents a split. For early fractions, ComENet and TFD2SimRefMCS rankers retrieve a higher rate of successful docking than bioactivity-unaware baselines

We next assessed the selection of highest-ranked conformers by AtNNs in rigid-ligand docking, to validate if the early enrichment of bioactive-like conformations previously observed helps in a structure-based practical application. Using a limited number of conformers allows to accelerate rigid-ligand docking: since the runtime scales linearly with the number of docked conformers, then selecting a fraction of 1% of conformers represents a 100-fold speedup. For each PDBbind test set ligand, a fraction of the top-ranked conformers, ranked by AtNNs or baselines, were rigidly docked to their cognate protein using GOLD. Then, either the highest PLP score or the lowest ARMSD pose was chosen for each molecule, and the proportion of molecules having a docking pose with an ARMSD to the native pose lower than 2 Å was computed as a success criterion. On the random splits, rigid-ligand docking using all conformers (i.e., fraction = 100%) and selecting the highest score pose leads to a successful docking rate of 0.70 ± 0.01, on a par with flexible docking results that reaches a rate of 0.68 ± 0.01, as shown in Fig. 10A; Table 6. Selecting the highest score pose on the 1% top-ranked conformer fraction, ComENet and the TFD2SimRefMCS baseline lead to successful docking rates of 0.48 ± 0.02 and 0.52 ± 0.02 respectively, outperforming the bioactivity-unaware baselines, with for instance the CSD Probability leading to a successful docking rate 0.39 ± 0.02. As we increase the fraction of selected conformers, the difference between bioactivity-based and bioactivity-unaware methods decreases, i.e., at the 20% fraction, ComENet, TFD2SimRefMCS and CSD Probability show successful docking rates of 0.65 ± 0.02, 0.66 ± 0.01 and 0.60 ± 0.01. Thus, ranking conformations using ComENet or the bioactivity-based baseline leads to improved successful docking compared to bioactivity-unaware baselines for small fractions of selected conformers.

**Table 6:** Successful docking rates when selecting highest PLP score poses (mean ± standard deviation)

Test set	Fraction (%)	Random order	CSD probability	Sage energy	ComENet	TFD2SimRefMCS	Flexible ligand docking
Random	1	0.34 ± 0.01	0.39 ± 0.02	0.37 ± 0.02	0.48 ± 0.02	0.52 ± 0.01	0.68 ± 0.01
	5	0.49 ± 0.01	0.5 ± 0.02	0.48 ± 0.02	0.56 ± 0.02	0.6 ± 0.01	
	20	0.6 ± 0.01	0.61 ± 0.01	0.61 ± 0.01	0.65 ± 0.02	0.66 ± 0.01	
	100	0.7 ± 0.01	0.7 ± 0.01	0.7 ± 0.01	0.7 ± 0.01	0.7 ± 0.01	
Scaffold	1	0.36 ± 0.03	0.39 ± 0.04	0.35 ± 0.02	0.44 ± 0.04	0.48 ± 0.03	0.68 ± 0.02
	5	0.5 ± 0.04	0.51 ± 0.03	0.47 ± 0.04	0.54 ± 0.04	0.54 ± 0.02	
	20	0.61 ± 0.03	0.61 ± 0.03	0.61 ± 0.03	0.62 ± 0.05	0.62 ± 0.02	
	100	0.69 ± 0.01	0.69 ± 0.01	0.69 ± 0.01	0.69 ± 0.01	0.69 ± 0.01	

On the scaffold splits, there is more variability between median success rate at the different fractions, such that only the TFD2SimRefMCS baseline is consistently outperforming the bioactivity-unaware baselines at the 1% fraction, as shown in Fig. 10B; Table 6, where ComENet, TFD2SimRefMCS and CSD Probability leads to successful docking rates of 0.43 ± 0.03, 0.48 ± 0.03 and 0.38 ± 0.02 respectively. Therefore, using bioactivity-based methods does not show utility beyond very small conformer fractions.

Docking algorithms are not always giving the highest docking score to the pose closest to the native pose [9]. To simulate the ideal case where the docking algorithm gives the highest score to the bioactive pose, we next re-evaluated the docking success rate by taking the poses with the lowest ARMSD instead of the highest scoring pose. Selecting all conformers shows a rigid-ligand docking success rate of 0.96 ± 0.00, outperforming flexible ligand docking having a rate of 0.86 ± 0.01, as shown in Fig. 10C; Table 7. Similar values are found for the scaffold split, as shown in Fig. 10D; Table 7. Hence, while the scoring function does not identify a successful docking pose as the highest scoring pose for 26% of docked ligands, rigid-ligand docking produces more successful poses than flexible docking when looking at all poses. The same trend of improved performance of bioactivity-based methods over bioactivity-unaware baselines are observed for the 1% and 5% fractions of the random splits, and 1% fraction of the scaffold split, i.e., ComENet, TFD2SimRefMCS and CSD Probability lead to successful rates of 0.54 ± 0.02, 0.57 ± 0.01 and 0.44 ± 0.02 for the 1% fraction of the random split, as shown in Fig. 10C; Table 7. Therefore, using the AtNN to rank conformers and select the top-ranked fractions helps retrieving bioactive-like conformations and subsequently successful docking, defined as achieving a pose close to the ligand-bound crystal structure.

**Table 7:** Successful docking rates when selecting lowest ARMSD score poses (mean ± standard deviation)

Test set	Fraction (%)	Random order	CSD probability	Sage energy	ComENet	TFD2SimRefMCS	Flexible ligand docking
Random	1	0.4 ± 0.01	0.44 ± 0.02	0.42 ± 0.02	0.54 ± 0.01	0.57 ± 0.01	0.86 ± 0.01
	5	0.67 ± 0.01	0.66 ± 0.02	0.62 ± 0.01	0.71 ± 0.01	0.73 ± 0.01	
	20	0.87 ± 0.01	0.84 ± 0.01	0.83 ± 0.01	0.86 ± 0.01	0.85 ± 0.01	
	100	0.96 ± 0.0	0.96 ± 0.0	0.96 ± 0.0	0.96 ± 0.0	0.96 ± 0.0	
Scaffold	1	0.42 ± 0.03	0.45 ± 0.04	0.4 ± 0.02	0.5 ± 0.04	0.53 ± 0.03	0.86 ± 0.01
	5	0.68 ± 0.02	0.67 ± 0.02	0.61 ± 0.03	0.68 ± 0.02	0.68 ± 0.02	
	20	0.85 ± 0.01	0.83 ± 0.01	0.83 ± 0.01	0.83 ± 0.01	0.82 ± 0.02	
	100	0.95 ± 0.01	0.95 ± 0.01	0.95 ± 0.01	0.95 ± 0.01	0.95 ± 0.01	

We finally performed the same analysis on specific test subsets corresponding to the 10 most represented enzyme classes, with successful rates of top score pose shown in Additional file 1: Fig. S7. For the 2.7.11, 3.4.21 and 3.4.23 classes, AtNN and TFD2SimRefMCS ranking is outperforming bioactivity-unaware baselines, while it is not the case for the other most represented enzyme classes. For instance, for the 3.4.21 class, the successful rate of ComENet selection at the 1% fraction (0.59 ± 0.07) is on average 84% greater than the one from the CSD Probability (0.32 ± 0.09). These results suggests that ranking methods leading to an early enrichment of bioactive-like conformations for over-represented enzyme classes give a higher rate of successful poses when used to select a limited set of input conformations.

### Selecting the AtNN highest-ranked conformers for pharmacophore searching leads to slightly higher hit rate compared to bioactivity-unaware baselines

**Fig. 11:**
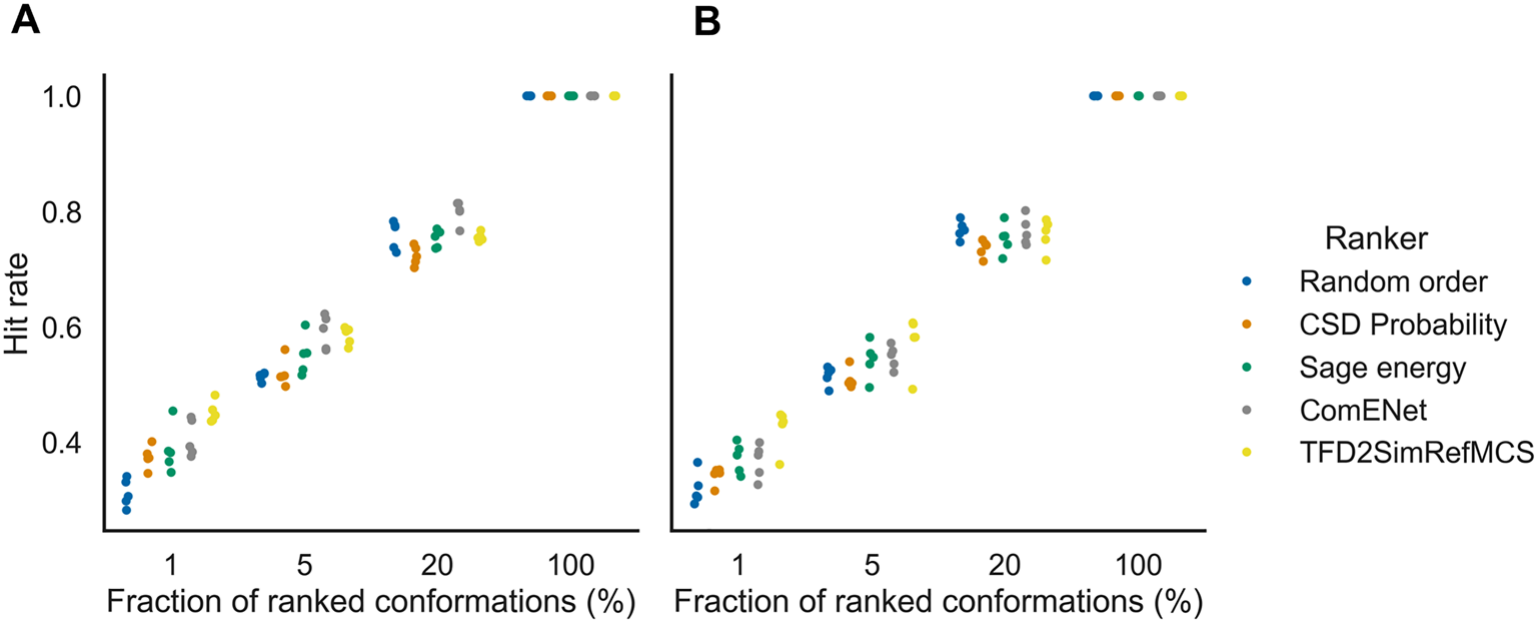
Pharmacophore searching hit rate selecting various fractions of conformers using different rankers, for the random (**A**) and scaffold (**B**) splits test sets. Each point represents a split. For early fractions (1% and 5%), TFD2SimRefMCS rankers shows a higher hit rate than bioactivity-unaware baselines, while for the 20% for the random split, ComENet shows a slightly higher hit rate

We next assessed the selection of highest-ranked conformers by AtNNs in pharmacophore searching, to validate if the early enrichment of bioactive-like conformations previously observed helps in a ligand-based application as much as in the rigid-ligand docking. For each PDBbind test set ligand, a pharmacophore query was elaborated from the bioactive conformation with up to 5 pharmacophoric features, and a fraction of the top-ranked conformers, ranked by AtNNs or baselines, was screened against this query. Only the molecules where at least one generated conformer matches with the query was kept for the analysis, to setup the 100% fraction to a 100% hit rate, in order to evaluate the hit rate in different fractions, shown in Fig. 11; Table 8. On the random splits, selecting the 1% top-ranked conformer fraction leads to a hit rate of 0.31 ± 0.02 for the Random order baseline. The CSD probability and Sage energy baselines outperform the Random order baseline with hit rates of 0.37 ± 0.02 and 0.39 ± 0.04 respectively. ComENet conformer ranking does not outperform the Sage energy baseline with a hit rate of 0.41 ± 0.03, while the TFD2SimRefMCS outperforms all baselines and the model with 0.45 ± 0.02. Selecting the 5% top-ranked conformer fraction leads to slightly higher hit rates for ComENet (0.59 ± 0.03) and the TFD2SimRefMCS baseline (0.58 ± 0.02) compared to the bioactivity-unaware baselines, ranging from 0.51 ± 0.01 to 0.55 ± 0.03. On the 20% fraction, ComENet slightly outperforms all baselines with a hit rate of 0.8 ± 0.02, compared to the best baseline being the TFD2SimRefMCS with a hit rate of 0.76 ± 0.01. On the scaffold split, the ComENet model shows smaller hit rates, on a par with the Sage energy baseline as shown in Fig. 11B; Table 8, while the only the TFD2SimRefMCS at the 1% fraction outperforms the bioactivity-unaware baselines. Therefore, ranking conformers with ComENet predictions leads to hit rates on a par or slightly higher than the bioactivity-based baseline, and higher than bioactivity-unaware baselines for selected conformer fractions above 1% for the random split.

**Table 8:** Hit rates of pharmacophore searching (mean ± standard deviation)

Test set	Fraction (%)	Random order	CSD probability	Sage energy	ComENet	TFD2SimRefMCS
Random	1	0.31 ± 0.02	0.37 ± 0.02	0.39 ± 0.04	0.41 ± 0.03	0.45 ± 0.02
	5	0.51 ± 0.01	0.52 ± 0.02	0.55 ± 0.03	0.59 ± 0.03	0.58 ± 0.02
	20	0.76 ± 0.02	0.72 ± 0.02	0.75 ± 0.02	0.8 ± 0.02	0.76 ± 0.01
	100	1.0 ± 0.0	1.0 ± 0.0	1.0 ± 0.0	1.0 ± 0.0	1.0 ± 0.0
Scaffold	1	0.32 ± 0.03	0.34 ± 0.02	0.37 ± 0.03	0.37 ± 0.03	0.42 ± 0.04
	5	0.52 ± 0.02	0.51 ± 0.02	0.54 ± 0.03	0.55 ± 0.02	0.57 ± 0.05
	20	0.77 ± 0.02	0.74 ± 0.01	0.75 ± 0.03	0.77 ± 0.02	0.76 ± 0.03
	100	1.0 ± 0.0	1.0 ± 0.0	1.0 ± 0.0	1.0 ± 0.0	1.0 ± 0.0

## Conclusions

In this study, we tackled the challenge of biasing conformer ensembles towards bioactive-like conformations by predicting the Atomic Root-Mean-Square Deviation to its closest bioactive conformation (ARMSD_bio_) for generated conformers of molecules using Atomistic Neural Network (AtNN) models. These predictions were used to rank the conformers of PDBbind test sets, generally obtaining a higher early enrichment of bioactive-like conformations and impoverishment of non-bioactive conformations than the bioactivity-unaware baselines. The early enrichment performances of AtNNs were comparable to a bioactivity-based baseline that uses Torsion Fingerprint Deviation to the Maximum Common Substructure to the closest training molecule (TFD2SimRefMCS). This early enrichment was consistently observed for the most represented protein target classes in PDBbind such as enzymes, where the training set often contained similar ligands to test ligands with large MCS. Training and test ligands matched by MCS torsion angles showed similar AtNN predicted values, suggesting that AtNN memorizes bioactive 3D arrangement of substructures, even though it struggles in specific cases, for example when the two structures differ by halogen atom positions or if the training set molecule is bigger. Finally, ranking using AtNN leads to higher successful docking rates than bioactivity-unaware baselines when only a limited number of conformers per molecule is used for rigid-ligand re-docking of PDBbind, and higher hit rates in pharmacophore searching.

In the context of reduction of conformations to test in virtual screening methods such as the presented rigid-docking procedure or pharmacophore elaboration and searching, the results obtained here suggest that selecting top-ranked conformations by AtNNs present similar results to MCS matching to similar molecules in a reference set while avoiding similarity or MCS computation, hence reducing computational time required. While the TFD2SimRefMCS baseline is directly interpretable by finding similar conformers (i.e., low TFD) of the MCS in the training set, it was found in our work to be two to three times slower than the AtNNs at test time (i.e., AtNNs require around two hours to train of a NVIDIA RTX 3080 GPU), and scales with the size of the training set. We therefore recommend using the AtNNs for conformer ranking if users have access to a GPU, and if the training set is large (i.e., at least in the order of tens of thousands) and contains ligands of proteins similar to the protein for which new ligands are screened.

The main limitation of AtNN ranking is the limited applicability to new scaffolds, as shown by the reduced ranking performances shown in the scaffold split. AtNNs are better at ranking conformations of molecules which have structurally similar molecules in the training set, and molecules with different scaffolds represent unseen chemical space. Also, AtNNs ranking performances were only consistently higher than bioactivity-unaware baselines for the most represented protein classes. Therefore, in practice, we recommend using AtNNs for known proteins for which there is sufficient ligand data available. This can be the case in initial conformation generation when in the training dataset there is a diversity of ligands with various scaffolds present, or when supporting lead optimization, where similar ligands are designed and therefore are expected to share similar binding poses for the common substructure.

The presented AtNN modelling approach only takes the ligand conformation as input, without explicitly incorporating knowledge of the protein target. However, ligands may bind to different proteins in different bioactive conformations. The model is trained to predict a single value that is the ARMSD to the closest bioactive conformation at training time; only one value is therefore predicted during evaluation, while it might be desirable to have one specific value per target. Potential improvements of current work include incorporating protein representations in the model for target-specific ARMSD_bio_ prediction. Also, the current AtNN methods have been repurposed from computational physics tasks (e.g., energy prediction), and their implementation could be adapted to the task at hand, for instance by implementing a global node in the graph neural network. Finally, the current work has been performed on conformers generated by the CSD conformer generator, which is based on crystal structure data. It is unsure whether the current approach is fully applicable to conformers generated with other methods, as the bond distances, valence angles, and sampled torsion angle profiles are not identical between conformer generators.

Overall, the approach presented here uses AtNNs to bias conformer ensemble towards bioactive-like conformations, representing an opportunity to accelerate conformation-seeded virtual screening techniques and other approaches where knowledge of a bioactive conformation is required.

### Supplementary Information

**Additional file 1.** It contains additional details on methods (e.g., manual PDBbind corrections, model parameters) and analysis on model regression performances, ChEMBL or ENZYME protein class specific performances.

## Data Availability

Ensembles of bioactive conformations and generated conformers, data splits, processed Pytorch Geometric dataset, RMSD computation and training logs are available on figshare: https://figshare.com/articles/dataset/Data_for_Applying_atomistic_neural_networks_to_bias_conformer_ensemble_towards_bioactive-like_conformations/23580267.  Pre-trained models are available on figshare: https://figshare.com/articles/dataset/Pretrained_atomistic_neural_networks/23586240.  Code to reproduce this work is available on GitHub: https://github.com/bbaillif/bioactive_conformation_predictor.

## Abbreviations

ARMSD: Atomic Root-Mean Square Deviation
ARMSD_bio_: ARMSD to the bioactive conformation
AtNN: Atomistic neural network
BEDROC: Boltzmann-Enhanced Discrimination of the Receiver Operating Characteristic
BEDROC_bio-like_: BEDROC of bioactive-like conformations
BEDROC_non-bio_: BEDROC of non-bioactive conformations
MCS: Maximum common substructure
SASA: Solvent accessible surface area
RGyr: Radius of gyration
TFD: Torsion fingerprint deviation
