# Supplementary material for: Applying atomistic neural networks to bias conformer ensembles towards bioactive-like conformations
Source: J Cheminform. 2023 Dec 21;15:124. doi: 10.1186/s13321-023-00794-w (PMC10740246; doi:10.1186/s13321-023-00794-w)
Supplement: Supplementary file 1 — Additional file 1. It contains additional details on methods (e.g., manual PDBbind corrections, model parameters) and analysis on model regression performances, ChEMBL or ENZYME protein class specific performances. [file 13321_2023_794_MOESM1_ESM.docx]

# Manual correction of PDBbind ligand names

'A' : 'AMP', # in 5o1u (was done in PDB)

'MAM' : 'MMA', # in 1ws5 (was done in PDB)

'GLB' : 'GAL', # in 1pum and 1oko (was done in PDB)

'BAR' : 'TSA', # in 2cht (was done in PDB)

'BAM' : 'BEN', # was done in PDB for the 8 ids that are in PDBbind

'I6P' : 'IHP', # in 5ijj and 5ijp (was done in PDB)

'U' : 'U5P', # in 1loq and 3gd1 (not done in PDB, but corresponds to single UMP)

'0IW' : 'D1R', # in 3qsd (not done in PDB, but 0IW is a "biologically interesting molecule" but is same as D1R in PDB)

# SQL query for ChEMBL target data extraction

SELECT accession, component_synonym, protein_class_desc

FROM component_sequences c

JOIN component_class d ON c.component_id = d.component_id

JOIN protein_classification e ON d.protein_class_id = e.protein_class_id

JOIN component_synonyms f ON c.component_id = f.component_id

WHERE f.syn_type = 'GENE_SYMBOL'

# Default parameters for atomistic neural networks

SchNet: 128 hidden embedding size, 6 interaction blocks and 50 radial basis function gaussians for the interatomic distance embeddings, 128 filters, cut-off of 10 A, maximum 32 neighbours.

DimeNet++: 128 hidden embedding size, 6 interaction blocks, 64 intermediate embedding size, 8 basis embedding size, 256 out embedding channels, 7 spherical basis and 6 radial basis, cutoff of 5 A, 32 maximum neighbours, envelope exponent of 5.

ComENet: Cut-off of 8 A, 4 layers, 256 hidden channels, 64 middle channels, 3 radial and 2 spherical basis

# Ligand-target distribution analysis

The distribution of number of bioactive conformations per ligand is shown in Figure S1A. There were 1264 ligands having more than one bioactive conformation in PDBbind, with a maximum of 79 conformations for ADP. We also looked at the distribution of the number of entries for each protein based on its Uniprot accession, shown in Figure S1B. There are 1412 proteins having more than one ligand in PDBbind, with a maximum of 363 for P00918 (carbonic anhydrase). There are a lot of seemingly repeated experiments in PDBbind, where a ligand is bound to the same protein (often a mutated version), leading to very similar conformations for the same ligand. The distribution of ARMSD between bioactive conformations (of identical molecules) is represented in Figure S1C, showing a substantial amount of ARMSD lower than 1. To assess this effect, we counted the number of ligands that had more than one conformation cluster as defined by the scipy fcluster algorithm with a 1 angstrom (Å) maximum overlay ARMSD using the GetBestRMS RDKit function. The distribution of the number of clusters is shown in Figure S1D. There were 428 ligands with more than one cluster (representing 4% of the total ligands), with up to 47 clusters for the ATP ligand.


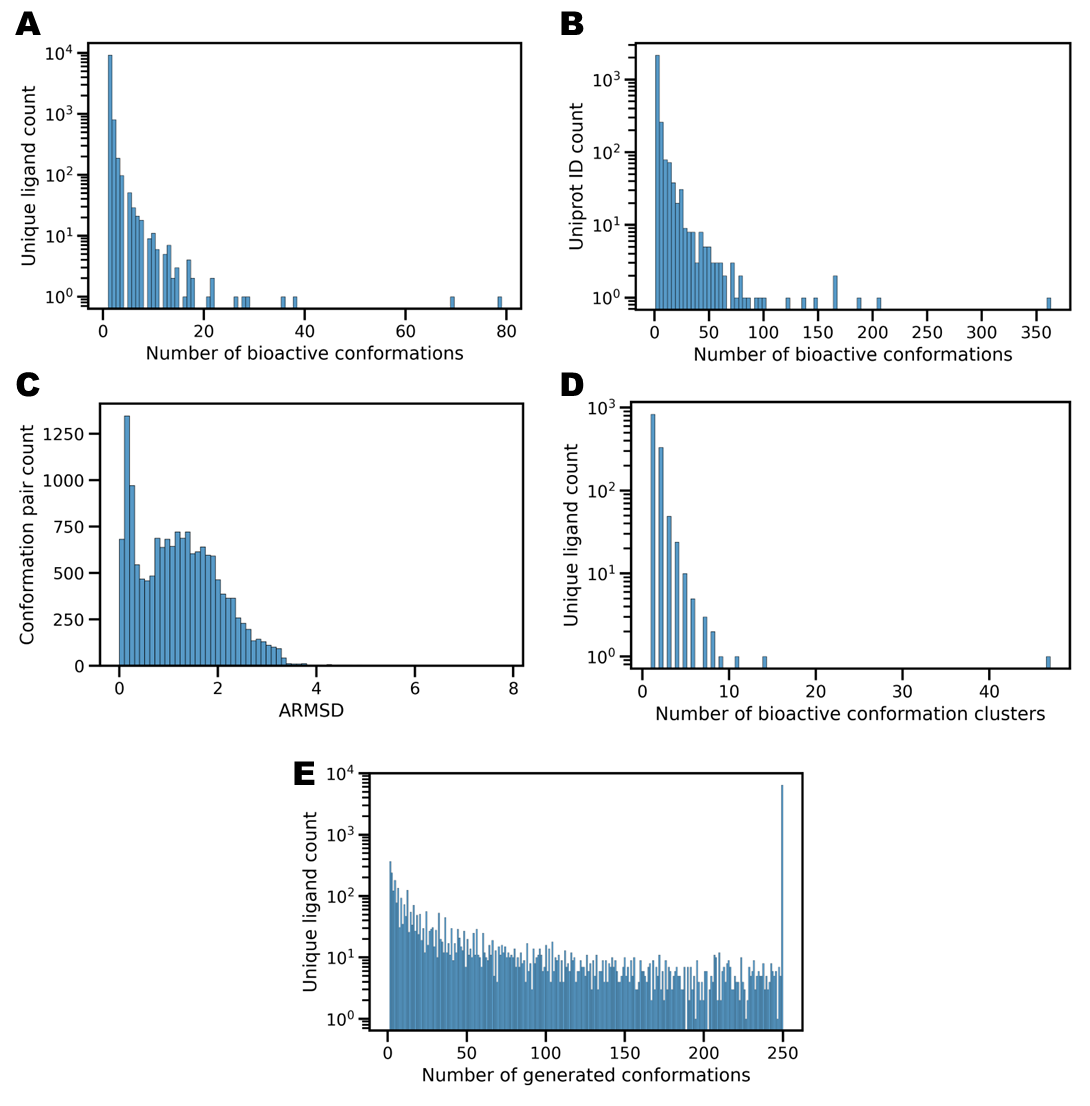


Figure S1: Metadata on PDBbind ligand and protein used in this study. A: Distribution of the number of bioactive conformations per unique ligand. B: Distribution of the number of ligand bioactive conformations per protein target. C: Distribution of the number of bioactive conformation clusters (ARMSD < 1 inside clusters) per unique ligand. D: Distribution of the ARMSD for each pair of bioactive conformation (same ligand). E: Distribution of the number of generated conformations per unique ligand.

# MCS size and TFD to the closest reference molecule


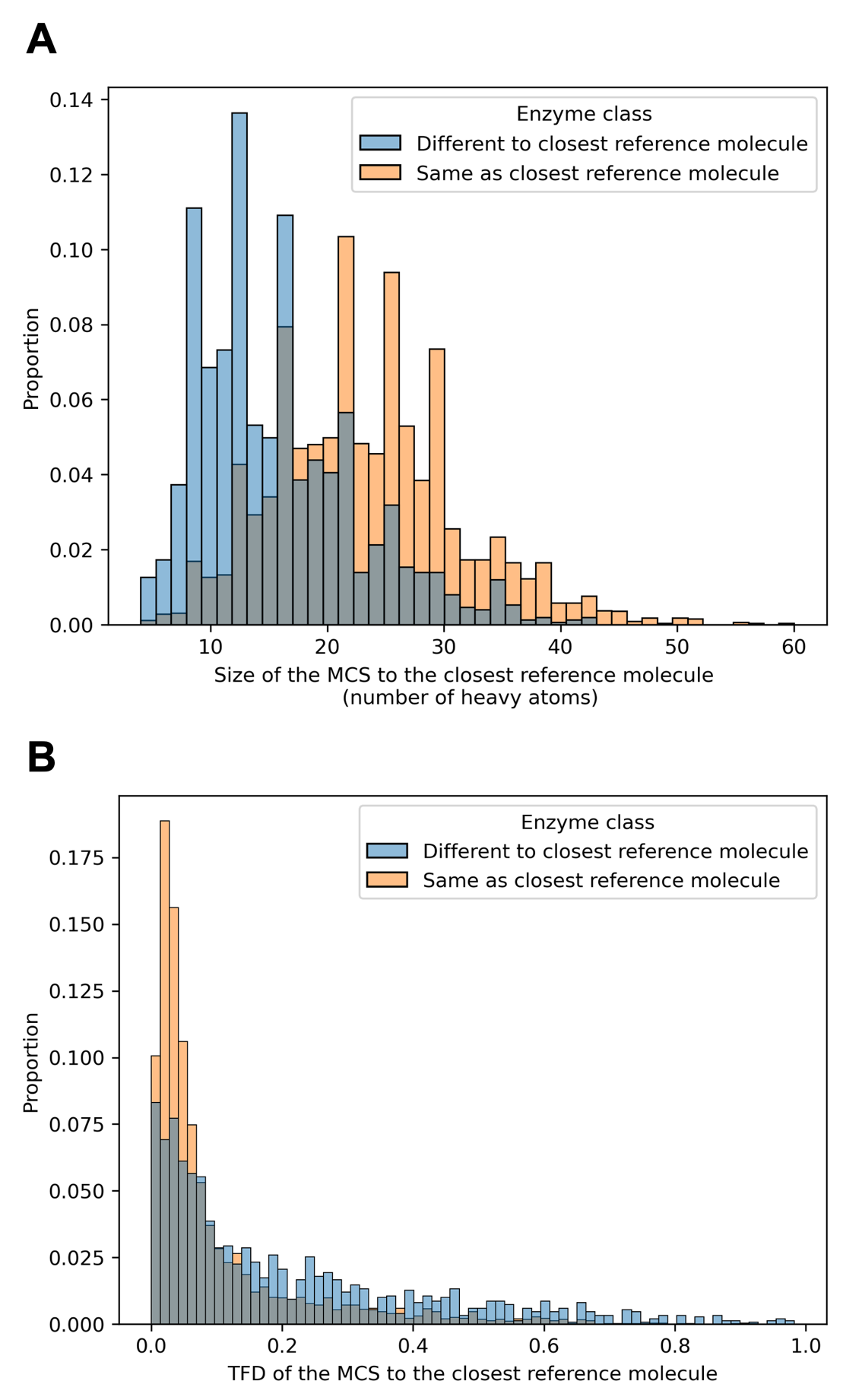


Figure S2 Distributions of the size and TFD of the MCS to the closest molecule in PDBbind. There are 4494 molecules for which the closest molecule is from the same enzyme class, while it was a different enzyme class for 1503 molecules. Molecules having the same class as the closest reference show higher MCS size and lower TFD on average.

# Regression results

The most expressive ComENet model leads to better ARMSD_bio_ with a lower RMSE and higher R^2^ compared to the least expressive SchNet model, as shown in Figure S3, suggesting that more expressive AtNNs better discriminate between bioactive-like conformations from non-bioactive conformations.


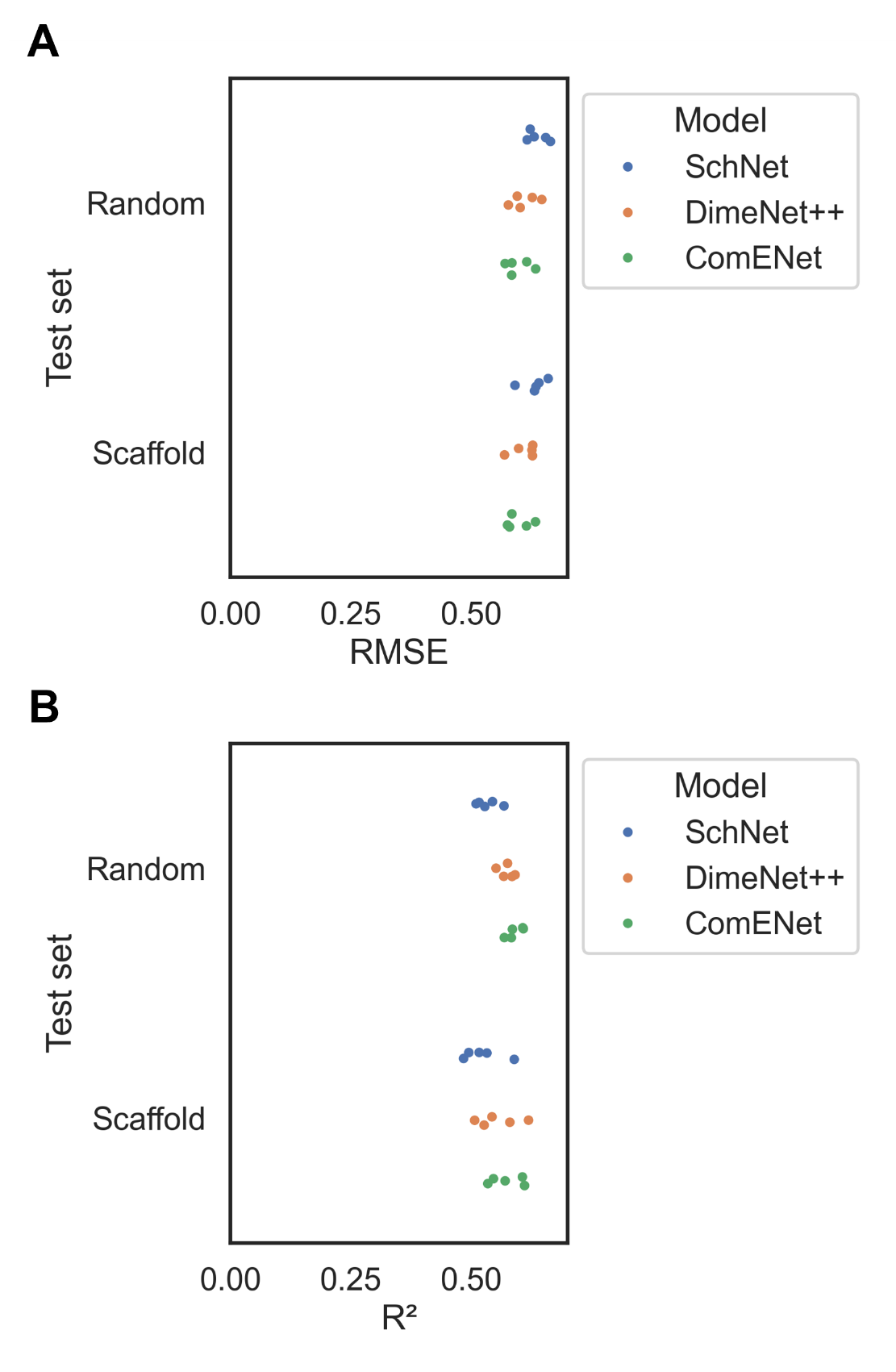


Figure S3: ARMSD_bio_ regression performances for the different AtNNs. A: RMSE and B: R2. Bar values are median values between splits, and error bars indicate the minimum and maximum values.

Table S1: Regression performances

| **Test set** | **Model** | **RMSE** | **R²** |
| --- | --- | --- | --- |
| **Random** | **SchNet** | 0.64 ± 0.02 | 0.53 ± 0.02 |
|  | **DimeNet++** | 0.61 ± 0.03 | 0.57 ± 0.02 |
|  | **ComENet** | 0.6 ± 0.03 | 0.59 ± 0.02 |
| **Scaffold** | **SchNet** | 0.63 ± 0.03 | 0.52 ± 0.04 |
|  | **DimeNet++** | 0.61 ± 0.03 | 0.56 ± 0.04 |
|  | **ComENet** | 0.6 ± 0.03 | 0.57 ± 0.03 |

# Protein class dependent results

Table S2: Median BEDROC of bioactive-like conformations for the top-10 ChEMBL protein classes

| **ChEMBL protein class** | **Number of complexes** | **Test set** | **Random order** | **CSD Probability** | **Sage energy** | **SchNet** | **DimeNet++** | **ComENet** | **TFD2SimRefMCS** |
| --- | --- | --- | --- | --- | --- | --- | --- | --- | --- |
| **enzyme** | 7322 | Random | 0.12 ± 0.02 | 0.17 ± 0.01 | 0.18 ± 0.02 | 0.22 ± 0.04 | 0.28 ± 0.02 | 0.3 ± 0.02 | 0.35 ± 0.01 |
|  |  | Scaffold | 0.13 ± 0.02 | 0.16 ± 0.02 | 0.18 ± 0.03 | 0.17 ± 0.05 | 0.22 ± 0.02 | 0.24 ± 0.04 | 0.29 ± 0.04 |
| **epigenetic regulator** | 814 | Random | 0.15 ± 0.02 | 0.17 ± 0.07 | 0.17 ± 0.06 | 0.14 ± 0.03 | 0.23 ± 0.11 | 0.24 ± 0.12 | 0.32 ± 0.11 |
|  |  | Scaffold | 0.16 ± 0.08 | 0.18 ± 0.07 | 0.2 ± 0.1 | 0.16 ± 0.06 | 0.21 ± 0.07 | 0.22 ± 0.09 | 0.25 ± 0.09 |
| **transcription factor** | 466 | Random | 0.1 ± 0.04 | 0.09 ± 0.02 | 0.18 ± 0.07 | 0.11 ± 0.04 | 0.24 ± 0.09 | 0.25 ± 0.12 | 0.26 ± 0.08 |
|  |  | Scaffold | 0.11 ± 0.02 | 0.16 ± 0.06 | 0.19 ± 0.05 | 0.13 ± 0.08 | 0.23 ± 0.11 | 0.2 ± 0.09 | 0.2 ± 0.07 |
| **cytosolic other** | 366 | Random | 0.1 ± 0.04 | 0.15 ± 0.05 | 0.14 ± 0.07 | 0.24 ± 0.12 | 0.21 ± 0.05 | 0.26 ± 0.11 | 0.5 ± 0.22 |
|  |  | Scaffold | 0.13 ± 0.06 | 0.16 ± 0.05 | 0.17 ± 0.09 | 0.13 ± 0.08 | 0.23 ± 0.13 | 0.34 ± 0.15 | 0.38 ± 0.22 |
| **unclassified** | 325 | Random | 0.13 ± 0.03 | 0.19 ± 0.07 | 0.19 ± 0.09 | 0.22 ± 0.07 | 0.3 ± 0.12 | 0.44 ± 0.17 | 0.38 ± 0.05 |
|  |  | Scaffold | 0.1 ± 0.06 | 0.1 ± 0.03 | 0.13 ± 0.06 | 0.08 ± 0.07 | 0.22 ± 0.09 | 0.31 ± 0.17 | 0.35 ± 0.17 |
| **ion channel** | 175 | Random | 0.09 ± 0.13 | 0.48 ± 0.45 | 0.45 ± 0.35 | 0.42 ± 0.41 | 0.19 ± 0.27 | 0.46 ± 0.42 | 0.18 ± 0.25 |
|  |  | Scaffold | 0.18 ± 0.07 | 0.27 ± 0.13 | 0.31 ± 0.23 | 0.24 ± 0.11 | 0.3 ± 0.21 | 0.18 ± 0.12 | 0.5 ± 0.3 |
| **membrane receptor** | 122 | Random | 0.16 ± 0.03 | 0.2 ± 0.07 | 0.2 ± 0.05 | 0.08 ± 0.05 | 0.2 ± 0.08 | 0.28 ± 0.14 | 0.19 ± 0.1 |
|  |  | Scaffold | 0.12 ± 0.07 | 0.21 ± 0.21 | 0.14 ± 0.1 | 0.03 ± 0.03 | 0.06 ± 0.06 | 0.09 ± 0.09 | 0.24 ± 0.37 |
| **secreted** | 116 | Random | 0.32 ± 0.33 | 0.38 ± 0.36 | 0.33 ± 0.24 | 0.3 ± 0.4 | 0.32 ± 0.4 | 0.38 ± 0.47 | 0.44 ± 0.35 |
|  |  | Scaffold | 0.22 ± 0.11 | 0.29 ± 0.23 | 0.32 ± 0.21 | 0.09 ± 0.04 | 0.22 ± 0.16 | 0.37 ± 0.35 | 0.31 ± 0.26 |
| **nuclear other** | 76 | Random | 0.14 ± 0.03 | 0.09 ± 0.05 | 0.18 ± 0.11 | 0.21 ± 0.06 | 0.27 ± 0.09 | 0.3 ± 0.32 | 0.6 ± 0.2 |
|  |  | Scaffold | 0.17 ± 0.07 | 0.15 ± 0.13 | 0.15 ± 0.11 | 0.14 ± 0.07 | 0.26 ± 0.19 | 0.31 ± 0.25 | 0.23 ± 0.08 |
| **auxiliary transport protein** | 52 | Random | 0.16 ± 0.16 | 0.1 ± 0.05 | 0.15 ± 0.14 | 0.16 ± 0.14 | 0.3 ± 0.31 | 0.24 ± 0.31 | 0.55 ± 0.37 |
|  |  | Scaffold | 0.29 ± 0.26 | 0.49 ± 0.37 | 0.44 ± 0.38 | 0.39 ± 0.35 | 0.44 ± 0.39 | 0.47 ± 0.34 | 0.67 ± 0.25 |


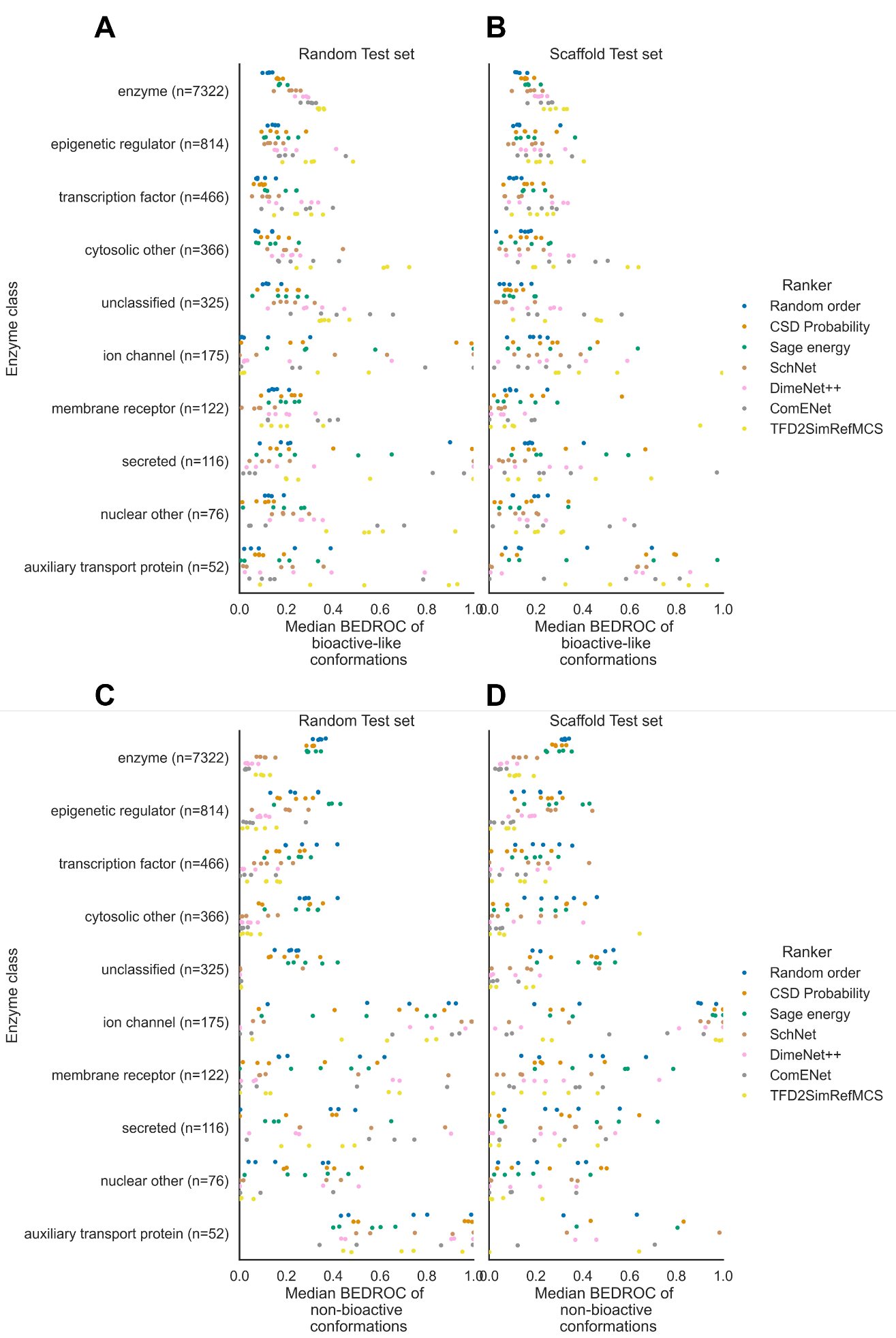


Figure S4: Protein class dependent median BEDROC of bioactive-like (A and B) and non-bioactive conformations (B and D) for the random (A and C) and scaffold split (B and D) test sets. AtNN and TFD2RefMCS are consistently outperforming the other data-agnostic baselines for enzymes only. Bar values are median values between splits, and error bars indicate the minimum and maximum values.


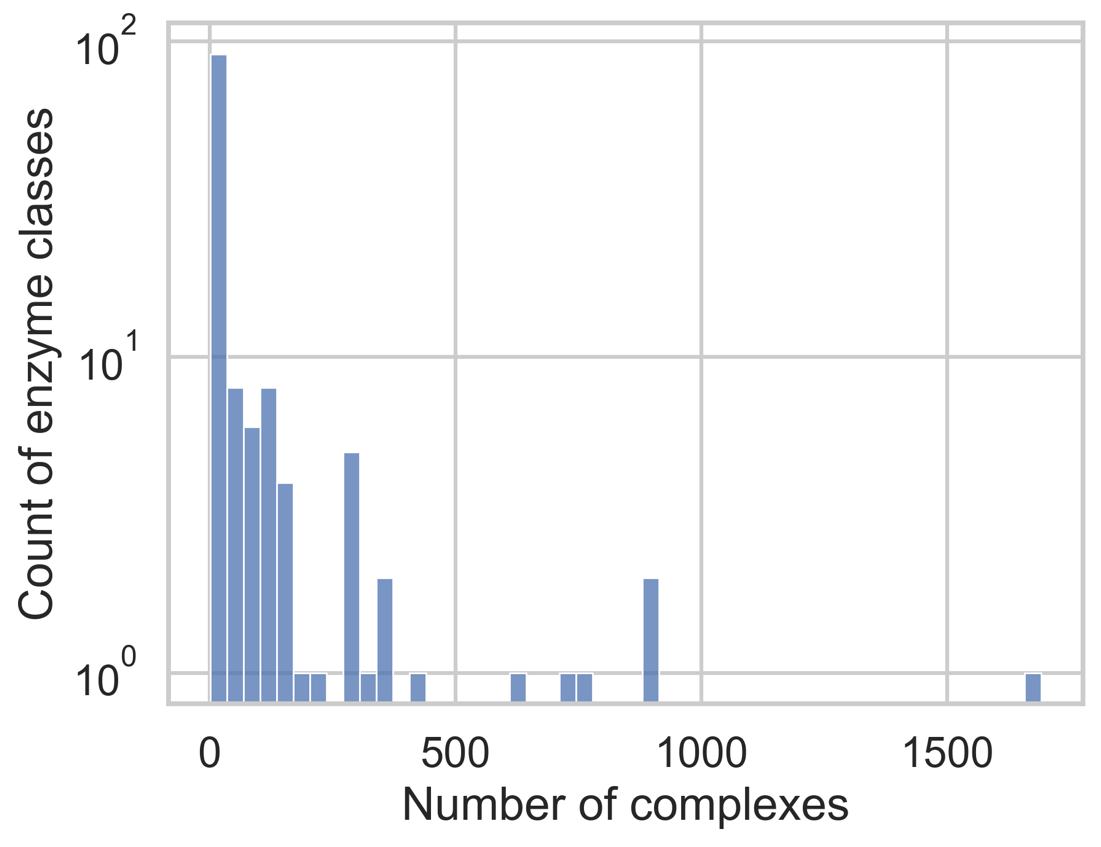


Figure S5: Histogram of the number of complexes per enzyme class.


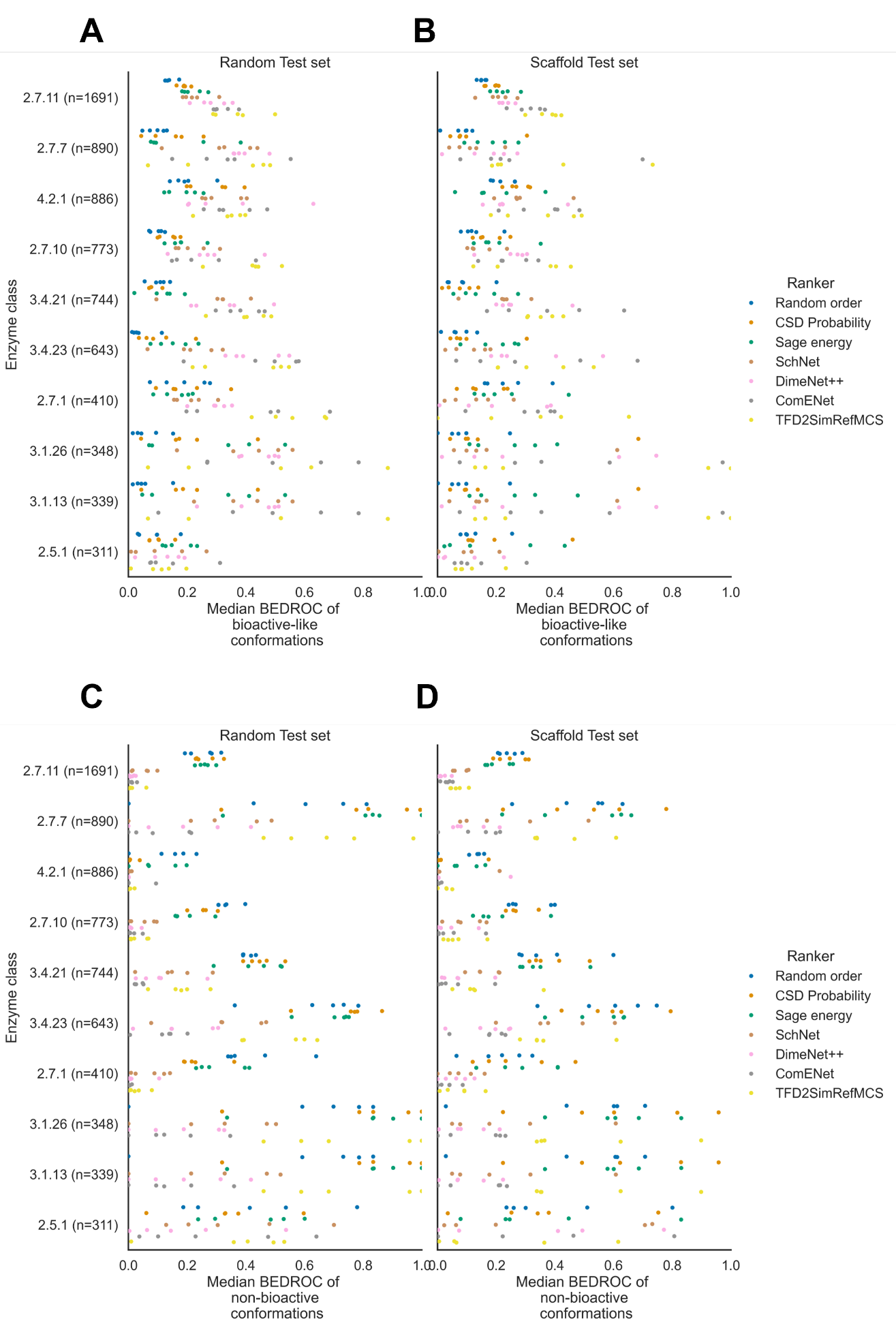


Figure S6: Enzyme class specific median BEDROC of bioactive-like (A and B) and non-bioactive conformations (C and D) for different ranking methods on the random (A and C) and scaffold (B and D) splits. Bar values are median values between splits, and error bars indicate the minimum and maximum values. AtNN or TFD2RefMCS methods outperforms bioactivity-unaware baselines for early enrichment of bioactive-like conformations on the 2.7.11, 3.4.21 and 3.4.23 classes only, and for early impoverishment of non-bioactive conformations on the 2.7.11, 2.7.10 and 3.4.21 classes.

Table S3: Median BEDROC of bioactive-like conformations for the top-10 ENZYME classes

| **ENZYME level 3** | **Number of complexes** | **Test set** | **Random order** | **CSD Probability** | **Sage energy** | **SchNet** | **DimeNet++** | **ComENet** | **TFD2SimRefMCS** |
| --- | --- | --- | --- | --- | --- | --- | --- | --- | --- |
| **2.7.11** | 1691 | Random | 0.14 ± 0.02 | 0.19 ± 0.02 | 0.22 ± 0.04 | 0.23 ± 0.05 | 0.29 ± 0.06 | 0.32 ± 0.04 | 0.37 ± 0.09 |
|  |  | Scaffold | 0.15 ± 0.01 | 0.19 ± 0.02 | 0.23 ± 0.04 | 0.21 ± 0.05 | 0.23 ± 0.02 | 0.31 ± 0.05 | 0.37 ± 0.05 |
| **2.7.7** | 890 | Random | 0.09 ± 0.03 | 0.15 ± 0.08 | 0.18 ± 0.14 | 0.32 ± 0.13 | 0.39 ± 0.05 | 0.33 ± 0.15 | 0.33 ± 0.18 |
|  |  | Scaffold | 0.08 ± 0.04 | 0.12 ± 0.11 | 0.18 ± 0.07 | 0.12 ± 0.07 | 0.17 ± 0.1 | 0.28 ± 0.24 | 0.35 ± 0.23 |
| **4.2.1** | 886 | Random | 0.2 ± 0.06 | 0.29 ± 0.08 | 0.19 ± 0.06 | 0.32 ± 0.07 | 0.34 ± 0.18 | 0.36 ± 0.09 | 0.34 ± 0.07 |
|  |  | Scaffold | 0.22 ± 0.04 | 0.27 ± 0.04 | 0.19 ± 0.12 | 0.3 ± 0.1 | 0.25 ± 0.11 | 0.36 ± 0.11 | 0.35 ± 0.14 |
| **2.7.10** | 773 | Random | 0.1 ± 0.02 | 0.14 ± 0.03 | 0.18 ± 0.05 | 0.22 ± 0.06 | 0.28 ± 0.12 | 0.25 ± 0.12 | 0.42 ± 0.1 |
|  |  | Scaffold | 0.13 ± 0.06 | 0.18 ± 0.05 | 0.21 ± 0.09 | 0.15 ± 0.06 | 0.25 ± 0.07 | 0.25 ± 0.08 | 0.31 ± 0.14 |
| **3.4.21** | 744 | Random | 0.1 ± 0.03 | 0.08 ± 0.02 | 0.12 ± 0.06 | 0.29 ± 0.12 | 0.33 ± 0.11 | 0.39 ± 0.07 | 0.4 ± 0.09 |
|  |  | Scaffold | 0.09 ± 0.07 | 0.08 ± 0.05 | 0.15 ± 0.09 | 0.21 ± 0.09 | 0.28 ± 0.11 | 0.38 ± 0.19 | 0.36 ± 0.05 |
| **3.4.23** | 643 | Random | 0.04 ± 0.04 | 0.11 ± 0.08 | 0.15 ± 0.07 | 0.21 ± 0.1 | 0.43 ± 0.09 | 0.45 ± 0.16 | 0.41 ± 0.16 |
|  |  | Scaffold | 0.07 ± 0.05 | 0.12 ± 0.11 | 0.2 ± 0.08 | 0.11 ± 0.07 | 0.32 ± 0.16 | 0.33 ± 0.25 | 0.34 ± 0.14 |
| **2.7.1** | 410 | Random | 0.19 ± 0.09 | 0.2 ± 0.1 | 0.16 ± 0.06 | 0.22 ± 0.06 | 0.29 ± 0.06 | 0.42 ± 0.2 | 0.56 ± 0.11 |
|  |  | Scaffold | 0.24 ± 0.09 | 0.16 ± 0.07 | 0.24 ± 0.13 | 0.13 ± 0.09 | 0.21 ± 0.17 | 0.3 ± 0.12 | 0.32 ± 0.25 |
| **3.1.26** | 348 | Random | 0.07 ± 0.06 | 0.21 ± 0.15 | 0.29 ± 0.21 | 0.41 ± 0.16 | 0.45 ± 0.06 | 0.49 ± 0.23 | 0.46 ± 0.33 |
|  |  | Scaffold | 0.09 ± 0.09 | 0.21 ± 0.27 | 0.25 ± 0.13 | 0.21 ± 0.23 | 0.35 ± 0.32 | 0.45 ± 0.34 | 0.49 ± 0.43 |
| **3.1.13** | 339 | Random | 0.06 ± 0.05 | 0.21 ± 0.15 | 0.28 ± 0.21 | 0.42 ± 0.13 | 0.42 ± 0.12 | 0.48 ± 0.27 | 0.44 ± 0.32 |
|  |  | Scaffold | 0.09 ± 0.09 | 0.21 ± 0.27 | 0.27 ± 0.15 | 0.22 ± 0.23 | 0.35 ± 0.32 | 0.45 ± 0.35 | 0.49 ± 0.43 |
| **2.5.1** | 311 | Random | 0.09 ± 0.06 | 0.1 ± 0.04 | 0.17 ± 0.05 | 0.11 ± 0.11 | 0.12 ± 0.07 | 0.14 ± 0.1 | 0.11 ± 0.07 |
|  |  | Scaffold | 0.13 ± 0.07 | 0.2 ± 0.15 | 0.19 ± 0.18 | 0.08 ± 0.09 | 0.08 ± 0.09 | 0.14 ± 0.1 | 0.12 ± 0.07 |


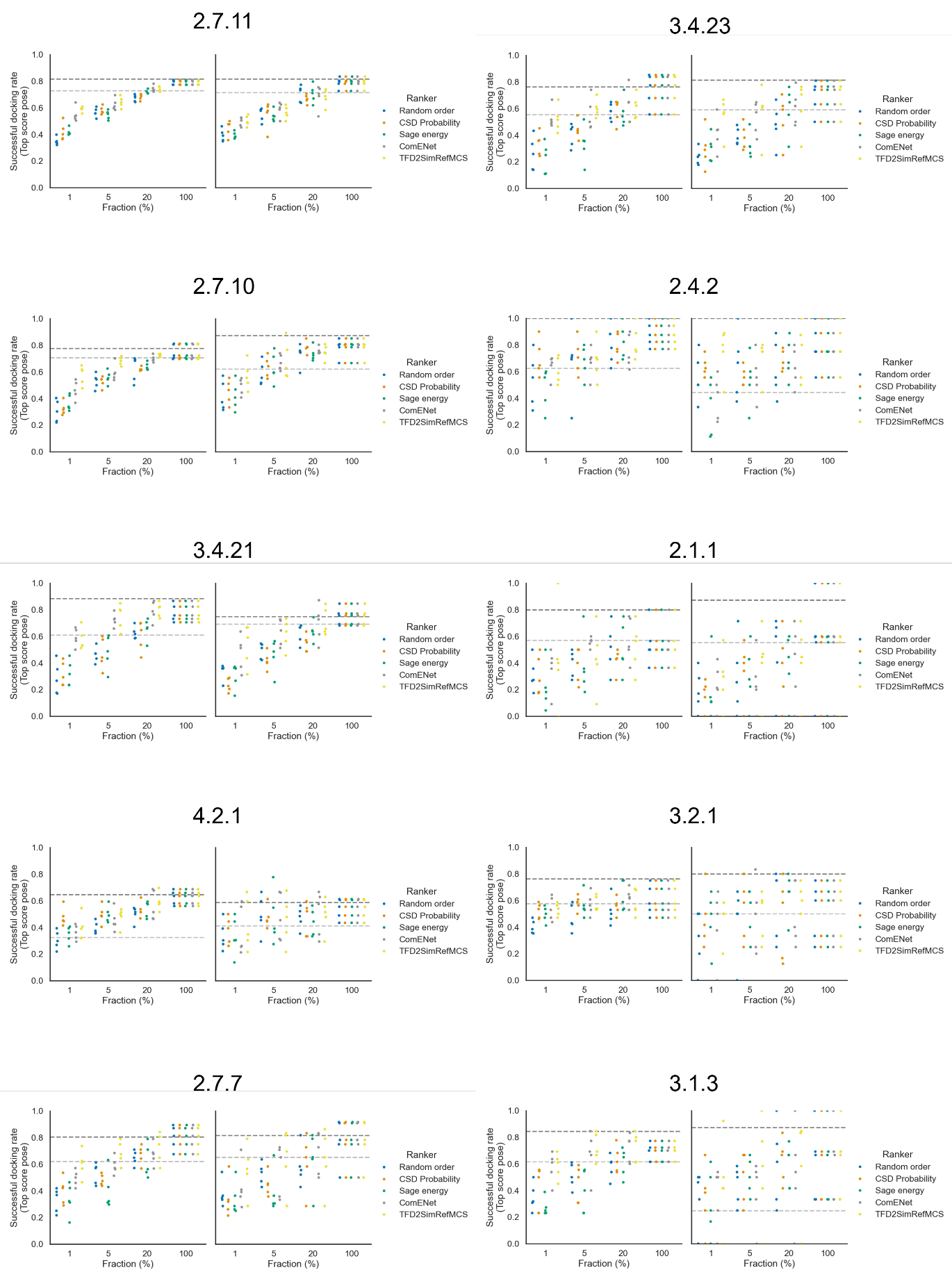


Figure S7: Successful docking rate for rigid-ligand redocking of PDBbind using variable fraction of input conformers per docked molecule, for the random splits (left part of each plot) and the scaffold splits (right part of each plot). Light and dark grey line respectively indicates flexible ligand docking minimum and maximum. Bar values are median values between splits, and error bars indicate the minimum and maximum values.
